# Supplementary material for: Controllable hierarchical self-assembly of porphyrin-derived supra-amphiphiles
Source: Nat Commun. 2019 Mar 28;10:1399. doi: 10.1038/s41467-019-09363-y (PMC6438973; doi:10.1038/s41467-019-09363-y)
Supplement: Supplementary file 1 — Supplementary Information [file 41467_2019_9363_MOESM1_ESM.pdf]

Supplementary Information

Controllable hierarchical self-assembly of porphyrin-derived supra-amphiphiles

Wang *et al.*

Supplementary Methods

TEM samples of 5 in THF and in aqueous solutions

In THF**:** A solution of **5** and a mixture solution of **5** and *n*-Bu_4NCl with a mole ratio of 1 : 4 at a concentration of 1 × 10^{-3} M in THF were prepared, respectively. These solutions were diluted to obtain solutions of **5** with and without Cl^- at a concentration of 1 × 10^{-4} M in THF. Drops of these solutions were cast on carbon-coated grids (Cu, 400 mesh) and dried naturally overnight before TEM photography.

In aqueous solution: 0.2 mL of a **5** solution at a concentration of 1 × 10^{-3} M in THF was mixed with 2 mL of deionized water. This mixture solution was stirred overnight until THF was volatilized completely. Drops of this aqueous solution of **5** were cast on carbon-coated grids (Cu, 400 mesh) and dried naturally for two days before TEM photography. Similarly, 0.2 mL of a mixture solution of **5** and *n*-Bu_4NCl with a mole ratio of 1 : 4 at a concentration of 1 × 10^{-3} M in THF was mixed with 2 mL of deionized water. This mixture solution was stirred overnight until THF was volatilized completely. Drops of this aqueous solution of **5** and *n*-Bu_4NCl were cast on carbon-coated grids (Cu, 400 mesh) and dried naturally for two days before TEM photography.

TEM samples of a mixture of 5 and 7 in aqueous solutions

A mixture solution of **5** and **7** with a mole ratio of 2 : 1 at a concentration of 1 × 10^{-3} M in THF was prepared by sonication for half an hour and filtration. To a certain amount of this $\mathbf{5_2}$**•7** solution, 4 equivalent *n*-Bu_4NCl was added to prepare a mixture solution of $\mathbf{5_2}$**•7** with Cl^- at a concentration of 1 × 10^{-3} M. After 0.2 mL of both the $\mathbf{5_2}$**•7** solutions with and without Cl^- were mixed with 2 mL of deionized water, respectively, the obtained mixture solutions were stirred overnight until THF was volatilized completely. Drops of these aqueous solutions were cast on carbon-coated grids (Cu, 400 mesh) and dried naturally for two days before TEM photography.

TEM samples of a mixture of 5 and 8 in aqueous solutions

A solution of **5** at a concentration of 2×10^{-3} M in THF was prepared, and a solution of **8** at a concentration of 1×10^{-3} M in toluene was prepared by sonication for half an hour and then filtration. The above two solutions were mixed to form a mixture solution of $\mathbf{5_2{\bullet}8}$ at a concentration of 1×10^{-3} M. To a certain amount of this $\mathbf{5_2{\bullet}8}$ solution, 4 equivalent n-Bu_4NCl was added to prepare a mixture solution of $\mathbf{5_2{\bullet}8}$ with Cl^-. After 0.2 mL of both the $\mathbf{5_2{\bullet}8}$ solutions with and without Cl^- were mixed with 2 mL of deionized water, respectively. Drops of these aqueous solutions were cast on carbon-coated grids (Cu, 400 mesh) and dried naturally for two days before TEM photography.

TEM samples of a mixture of 6 and 9 in aqueous solutions

A mixture solution of **6** and **9** with a mole ratio of 2 : 1 at a concentration of 1×10^{-3} M in THF was prepared by sonication for half an hour and filtration. To a certain amount of this $\mathbf{6_2{\bullet}9}$ solution, 4 equivalent n-Bu_4NCl was added to prepare a mixture solution of $\mathbf{6_2{\bullet}9}$ with Cl^- at a concentration of 1×10^{-3} M. After 2 mL of both the $\mathbf{6_2{\bullet}9}$ solutions with and without Cl^- were mixed with 2 mL of deionized water, respectively, the obtained mixture solutions were stirred overnight until THF was volatilized completely. Drops of these aqueous solutions were cast on carbon-coated grids (Cu, 400 mesh) and dried naturally for two days before TEM photography.

TEM samples of a mixture of 6 and 10 in aqueous solutions

A mixture solution of **6** and **10** with a mole ratio of 3 : 1 at a concentration of 1×10^{-3} M in THF was prepared by sonication for half an hour and filtration. To a certain amount of this $\mathbf{6_3{\bullet}10}$ solution, 4 equivalent n-Bu_4NCl was added to prepare a mixture solution of $\mathbf{6_3{\bullet}10}$ with Cl^- at a concentration of 1×10^{-3} M. After 2 mL of both the $\mathbf{6_3{\bullet}10}$ solutions with and without Cl^- were mixed with 2 mL of deionized water, respectively, the obtained mixture solutions were stirred overnight until THF was volatilized completely. Drops of these aqueous solutions were cast on carbon-coated grids (Cu, 400 mesh) and dried naturally for two days before TEM photography.

SEM samples of a mixture of 5 and 7 in aqueous solutions

A mixture solution of **5** and **7** with a mole ratio of 2 : 1 at a concentration of 1×10^{-3} M in THF was prepared by sonication for half an hour and filtration. To a certain amount of this $\mathbf{5_2{\bullet}7}$ solution, 4 equivalent *n*-Bu_4NCl was added to prepare a mixture solution of $\mathbf{5_2{\bullet}7}$ with Cl^- at a concentration of 1×10^{-3} M. After 0.2 mL of both the $\mathbf{5_2{\bullet}7}$ solutions with and without Cl^- were mixed with 2 mL of deionized water, respectively, the obtained mixture solutions were stirred overnight until THF was volatilized completely. Drops of these aqueous solutions were cast on monocrystallinesilicon, dried naturally for two days and sprayed gold before SEM photography.

SEM samples of a mixture of 5 and 8 in aqueous solutions

A solution of **5** at a concentration of 2×10^{-3} M in THF was prepared, and a solution of **8** at a concentration of 1×10^{-3} M in toluene was prepared by sonication for half an hour and then filtration. The above two solutions were mixed to form a mixture solution of $\mathbf{5_2{\bullet}8}$ at a concentration of 1×10^{-3} M. To a certain amount of this $\mathbf{5_2{\bullet}8}$ solution, 4 equivalent *n*-Bu_4NCl was added to prepare a mixture solution of $\mathbf{5_2{\bullet}8}$ with Cl^-. After 0.2 mL of both the $\mathbf{5_2{\bullet}8}$ solutions with and without Cl^- were mixed with 2 mL of deionized water, respectively. Drops of these mixture solutions were cast on monocrystallinesilicon and dried naturally until the mixture solutions were volatilized completely, then sprayed gold before SEM photography.

SEM samples of a mixture of 6 and 10 in aqueous solutions

A mixture solution of **6** and **10** with a mole ratio of 3 : 1 at a concentration of 1×10^{-3} M in THF was prepared by sonication for half an hour and filtration. To a certain amount of this $\mathbf{6_3{\bullet}10}$ solution, 4 equivalent *n*-Bu_4NCl was added to prepare a mixture solution of $\mathbf{6_3{\bullet}10}$ with Cl^- at a concentration of 1×10^{-3} M. After 2 mL of both the $\mathbf{6_3{\bullet}10}$ solutions with and without Cl^- were mixed with 2 mL of deionized water, respectively, the obtained mixture solutions were stirred overnight until THF was volatilized completely. Drops of these aqueous solutions were cast on

monocrystallinesilicon, dried naturally for two days and sprayed gold before SEM photography.

Preparation of AFM samples

A solution of **5** at a concentration of 2×10^{-3} M in THF was prepared, and a solution of **8** at a concentration of 1×10^{-3} M in toluene was prepared by sonication for half an hour and then filtration. The above two solutions were mixed to form a mixture solution of $\mathbf{5_2 \bullet 8}$ at a concentration of 1×10^{-3} M. After 0.2 mL of the $\mathbf{5_2 \bullet 8}$ solutions was mixed with 2 mL of deionized water, drops of the mixture solutions was cast on monocrystallinesilicon and dried naturally for two days before, then sprayed gold before AFM photography.

Measurement of CACs

Solutions of the amphiphiles **5** and **6** and the supra-amphiphiles at a concentration of 1×10^{-3} M in THF were prepared, respectively. Different volumes of deionized water were added to dilute the solutions to obtain the aqueous solutions with different concentrations at a range from 1×10^{-4} M to 1×10^{-7} M. The transmittances of the aqueous solutions were then measured by UV-vis spectroscopy, and the correlation between the concentration and transmittance was made at the point where the ultraviolet absorption wavelength was 430 nm.

Supplementary Figures

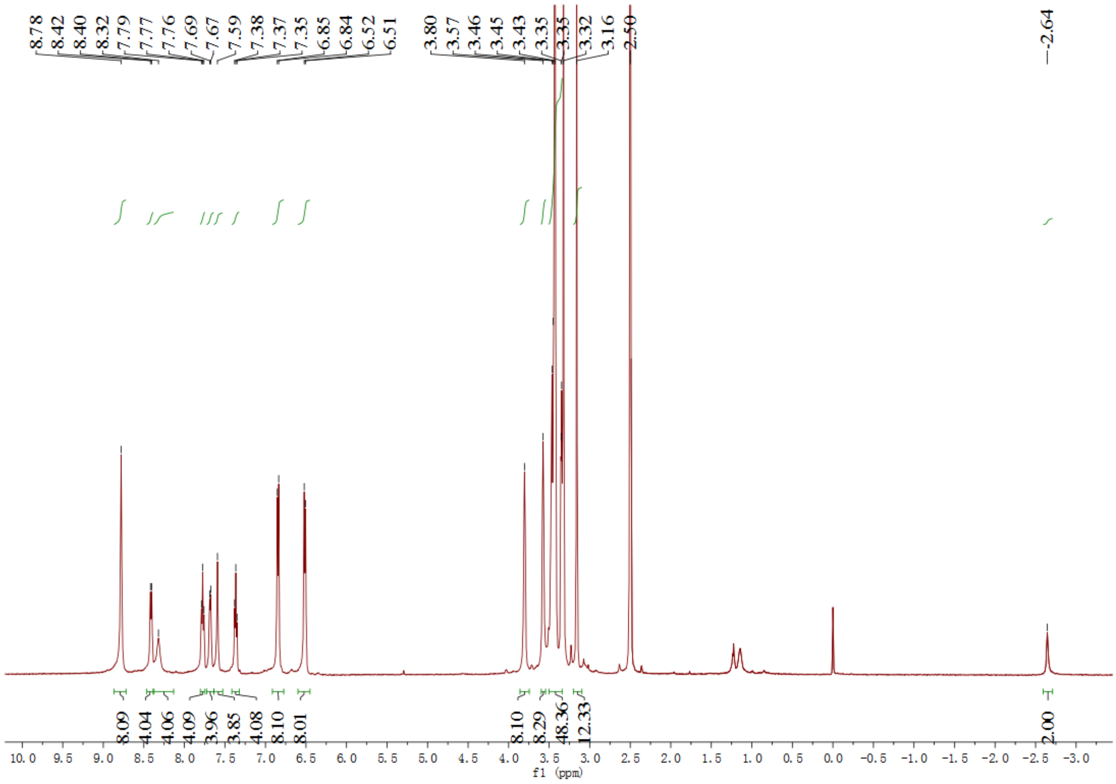

Supplementary Figure 1. ^{1}H NMR spectrum (500 MHz, DMSO-d_6, 22 ℃) of **5**.

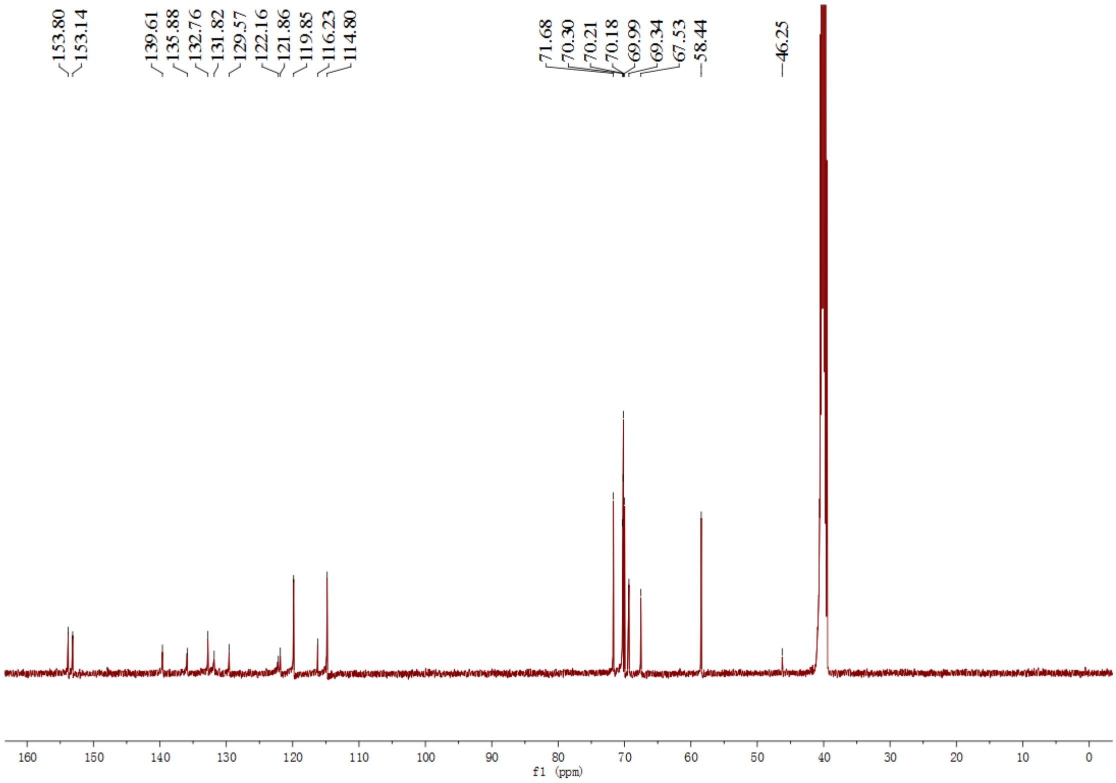

Supplementary Figure 2. ^{13}C NMR spectrum (500 MHz, DMSO-d_6, 22 ℃) of **5**.

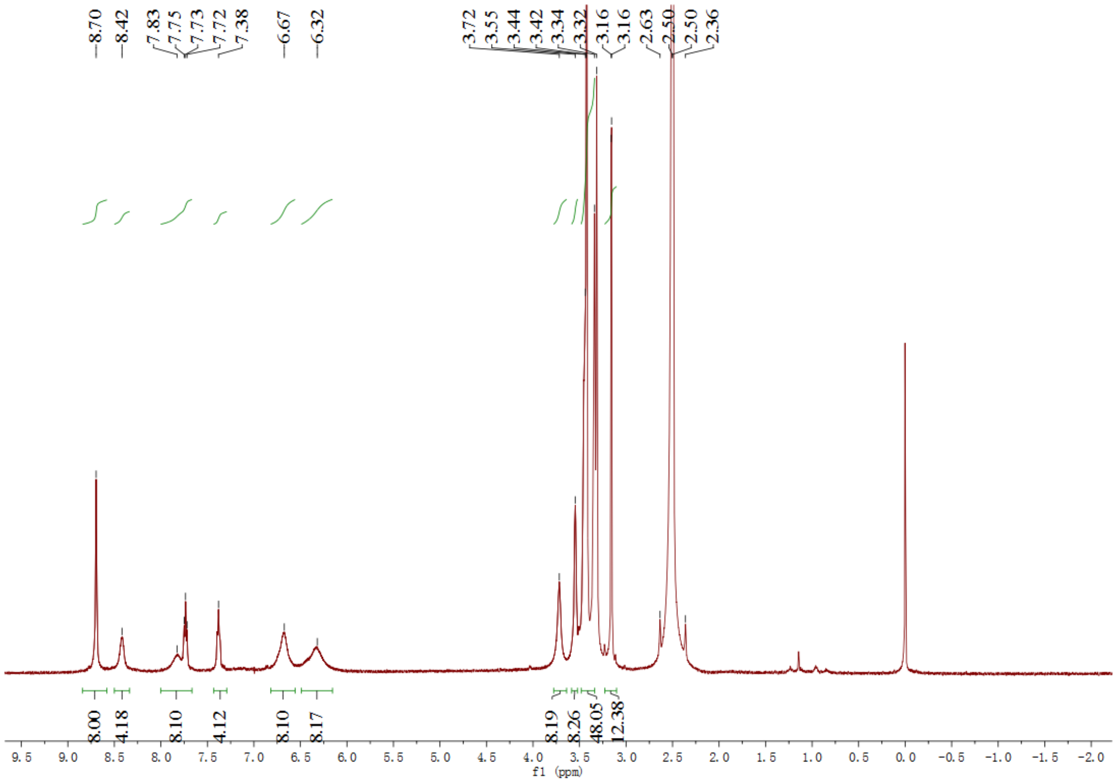

Supplementary Figure 3. ^{1}H NMR spectrum (500 MHz, DMSO-d_6, 22 ℃) of **6**.

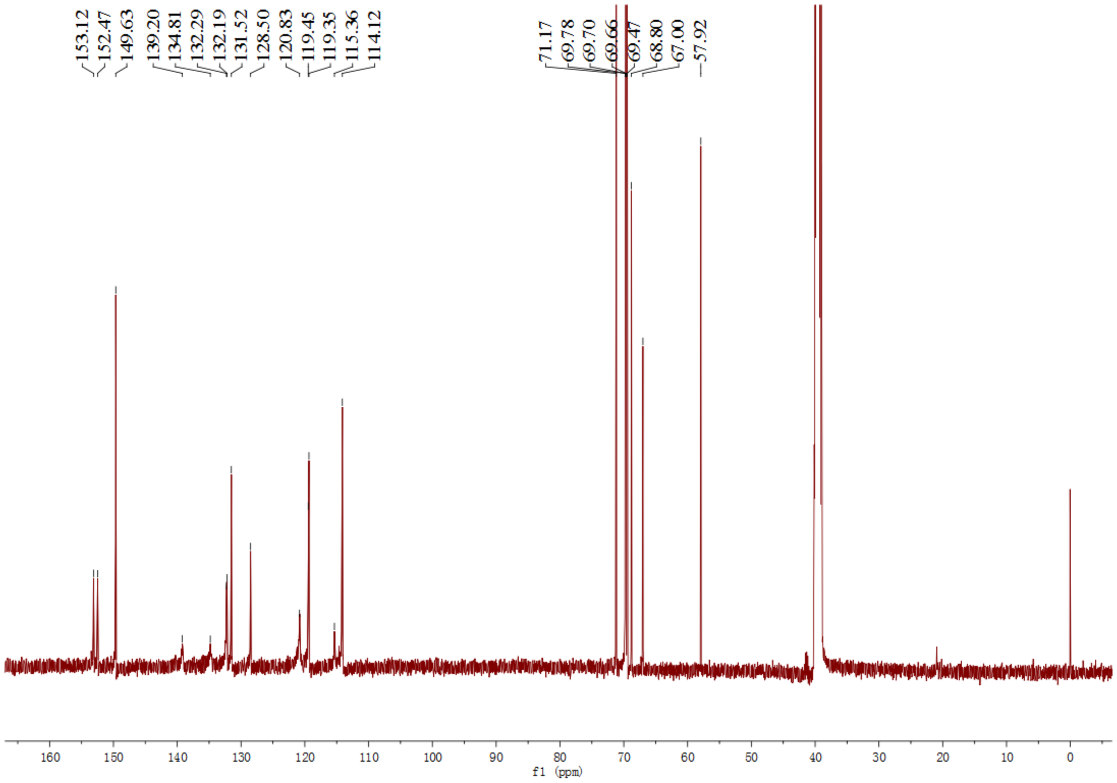

Supplementary Figure 4. ^{13}C NMR spectrum (500 MHz, DMSO-d_6, 22 ℃) of **6**.

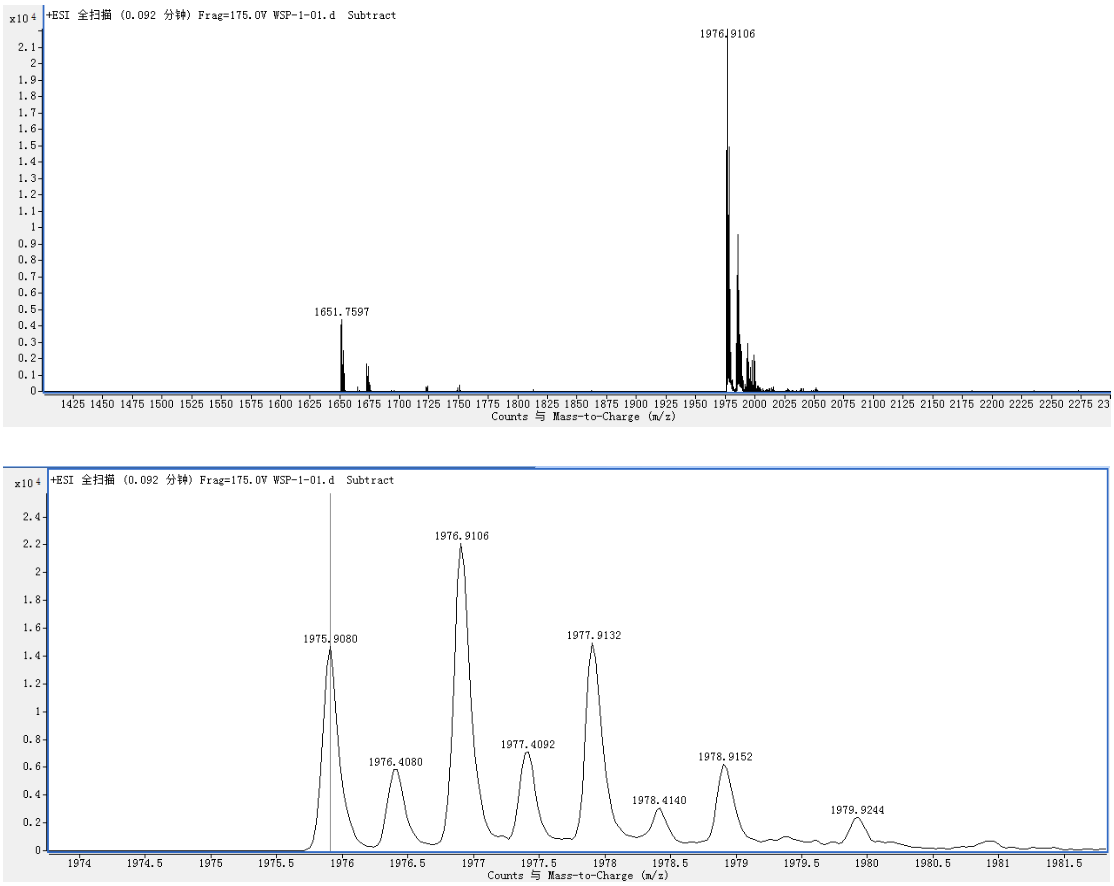

Supplementary Figure 5. Electrospray ionization mass spectrum of **5**. Calcd for $C_{108}H_{126}N_{12}O_{12}$ $[M + H]^+$: 1975.9008, Found:1975.9080, error 3.6 ppm.

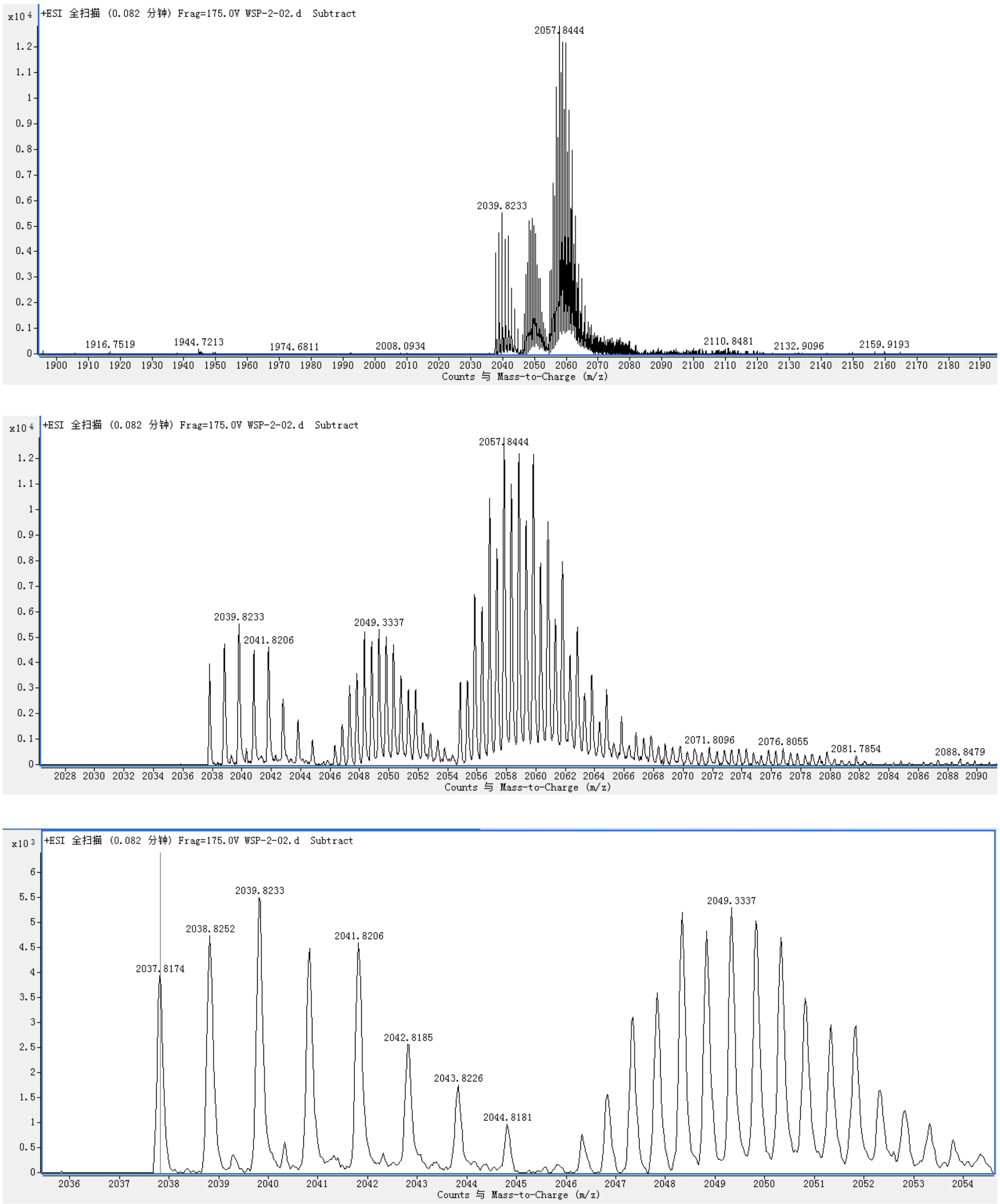

Supplementary Figure 6. Electrospray ionization mass spectrum of **6**. Calcd for $C_{108}H_{124}N_{12}O_{12}Zn$ $[M + H]^+$: 2037.8143, Found: 2037.8174, error 1.5 ppm.

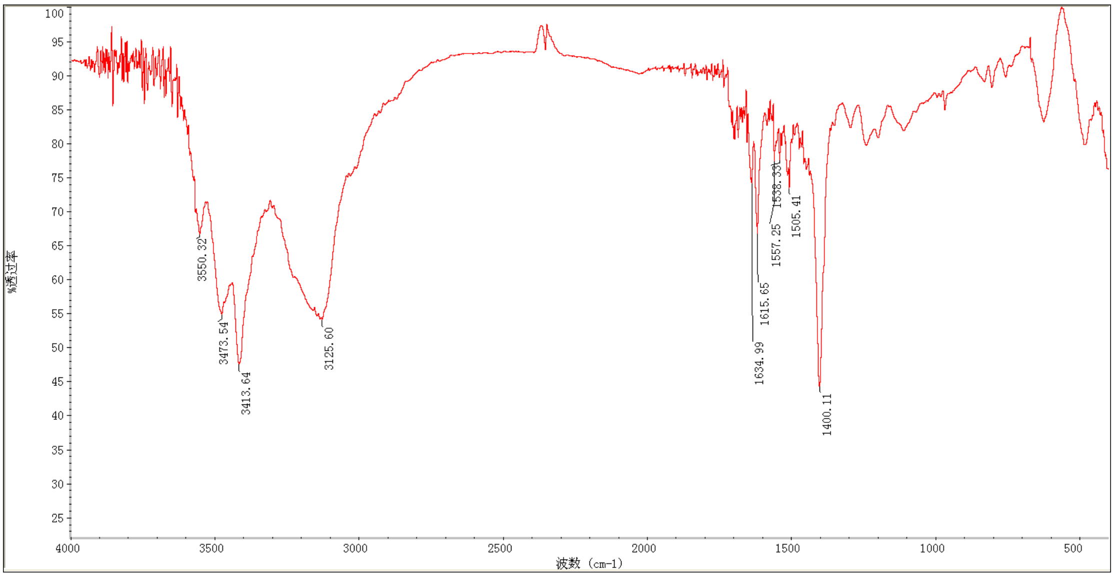

Supplementary Figure 7. FT-IR spectrum of **5.**

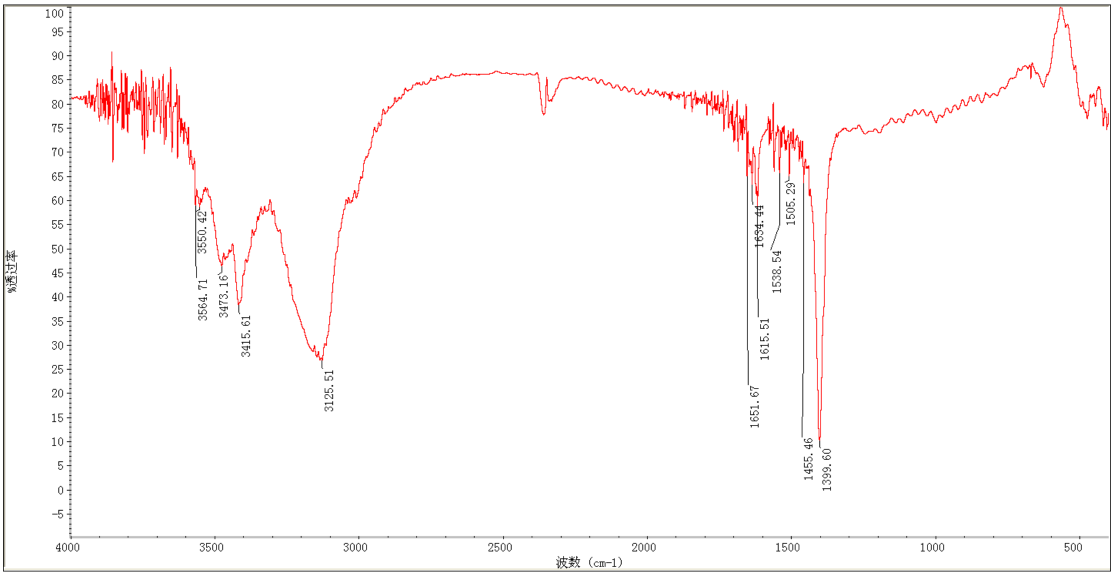

Supplementary Figure 8. FT-IR spectrum of **6.**

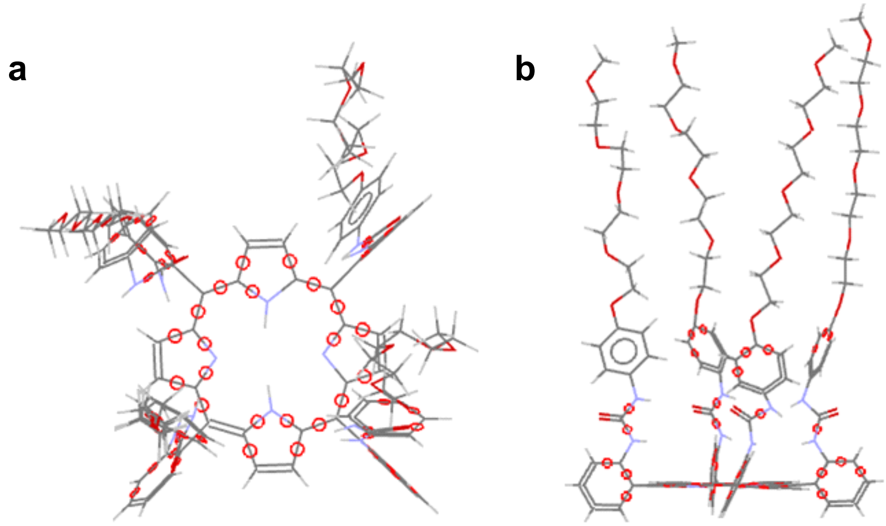

Supplementary Figure 9. Simulated molecular structure of **5**. **a** top view. **b** front view.

Molecular force field simulations were carried out to gain insight into the structural characteristics of the porphyrin-based heteroditopic amphiphile **5**. A 1.0 ns molecular dynamics simulation (AMBER force field) was used to equilibrate the molecule, and then the energies of the resulting structures were minimized to full convergence.

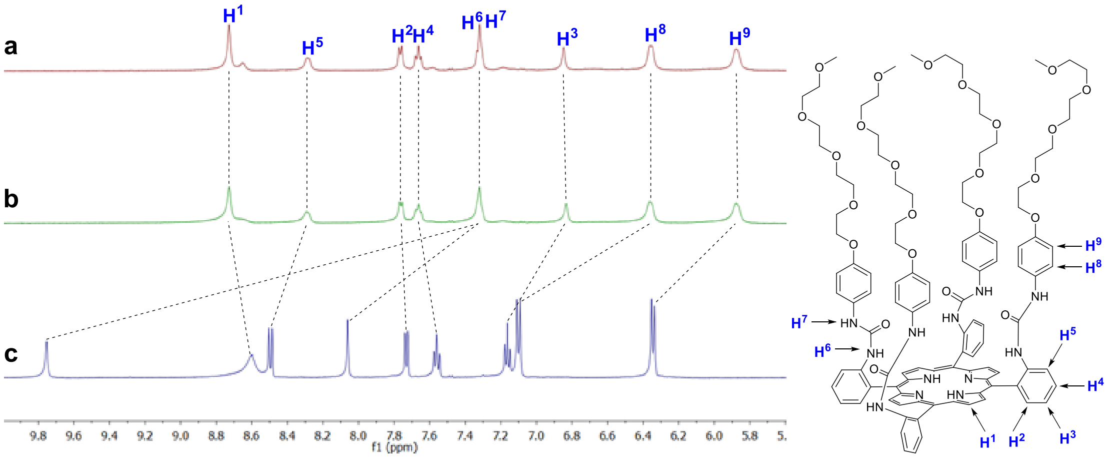

Supplementary Figure 10. Partial ^{1}H NMR (500 MHz, THF-d_8, 22 ℃) spectra. **a** A mixture of **5** and NaCl, after stirring for 10 h. **b** **5**. **c** A mixture of **5** and n-Bu_4NCl.

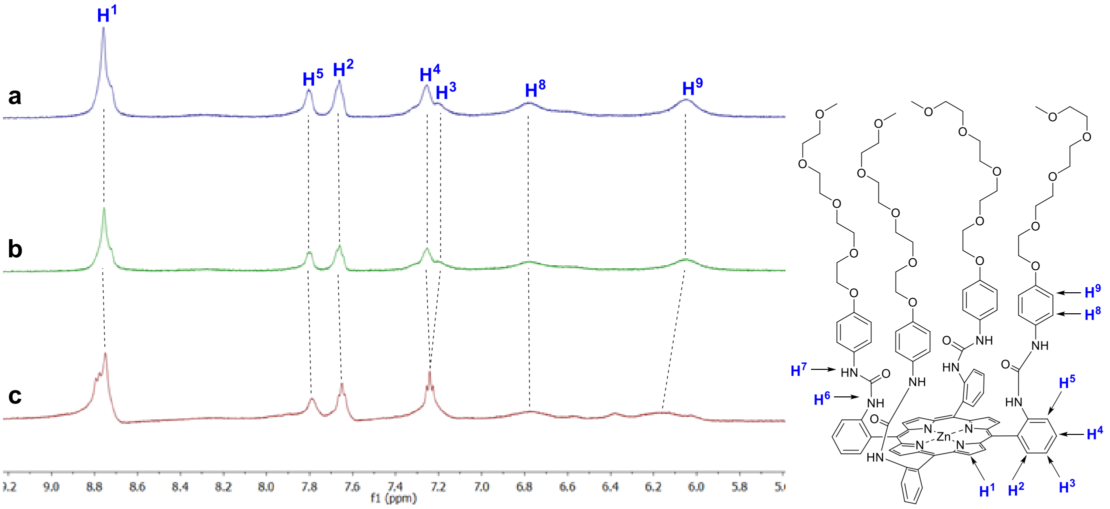

Supplementary Figure 11. Partial ^{1}H NMR (500 MHz, THF-d_8, 22 ℃) spectra. **a** A mixture of **6** and NaCl, after stirring for 10 h. **b** **6**. **c** A mixture of **6** and n-Bu_4NCl.

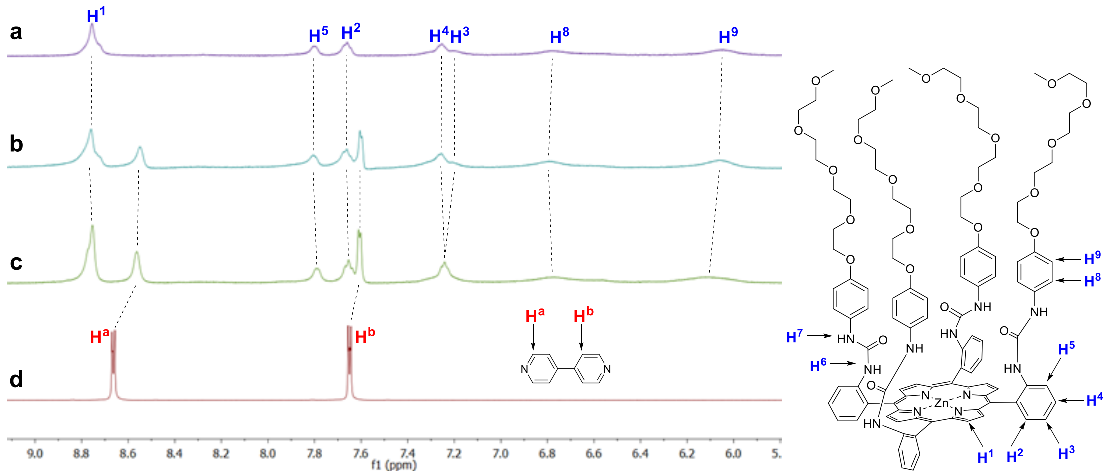

Supplementary Figure 12. Partial ^{1}H NMR (500 MHz, THF-d_8, 22 ℃) spectra. **a** **6**. **b** $\mathbf{6_2}$**•9**. **c** A mixture of $\mathbf{6_2}$**•9** and *n*-Bu_4NCl. **d** **9**.

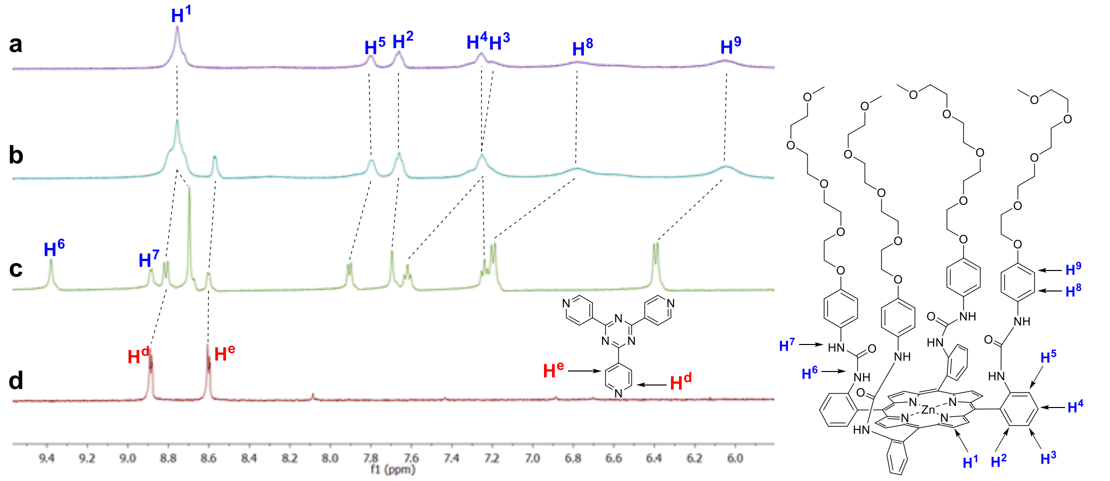

Supplementary Figure 13. Partial ^{1}H NMR (500 MHz, THF-d_8, 22 ℃) spectra. **a** **6**. **b** $\mathbf{6_3}$**•10**. **c** A mixture of $\mathbf{6_3}$**•10** and *n*-Bu_4NCl. **d** **10**.

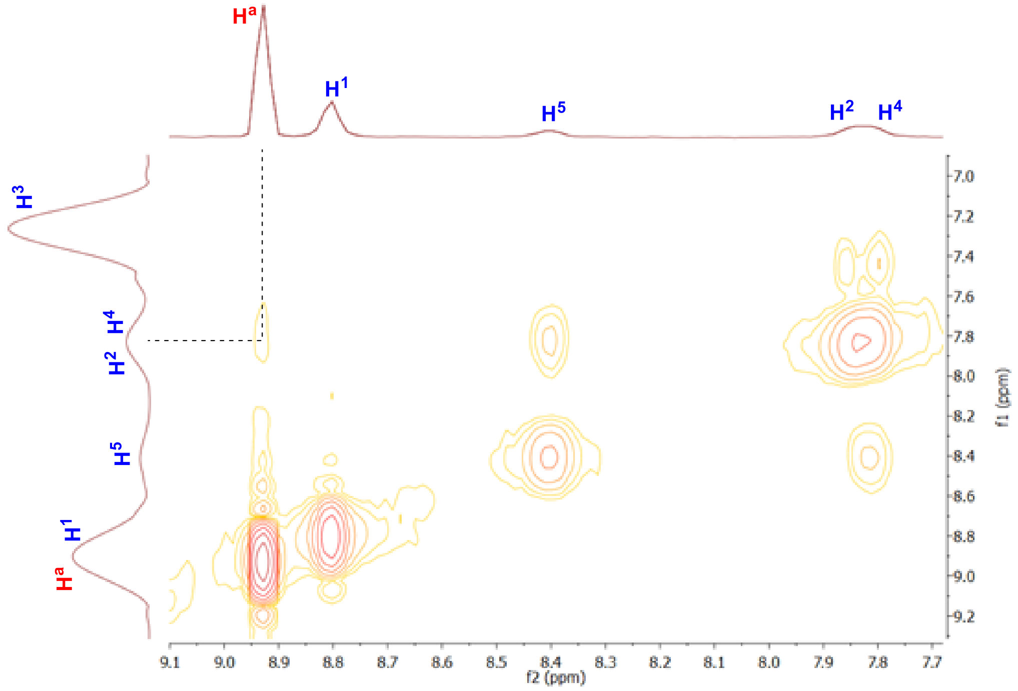

Supplementary Figure 14. NOESY spectrum (500 MHz, $CDCl_3$, 22 ℃) of a mixture of **5** and **7**.

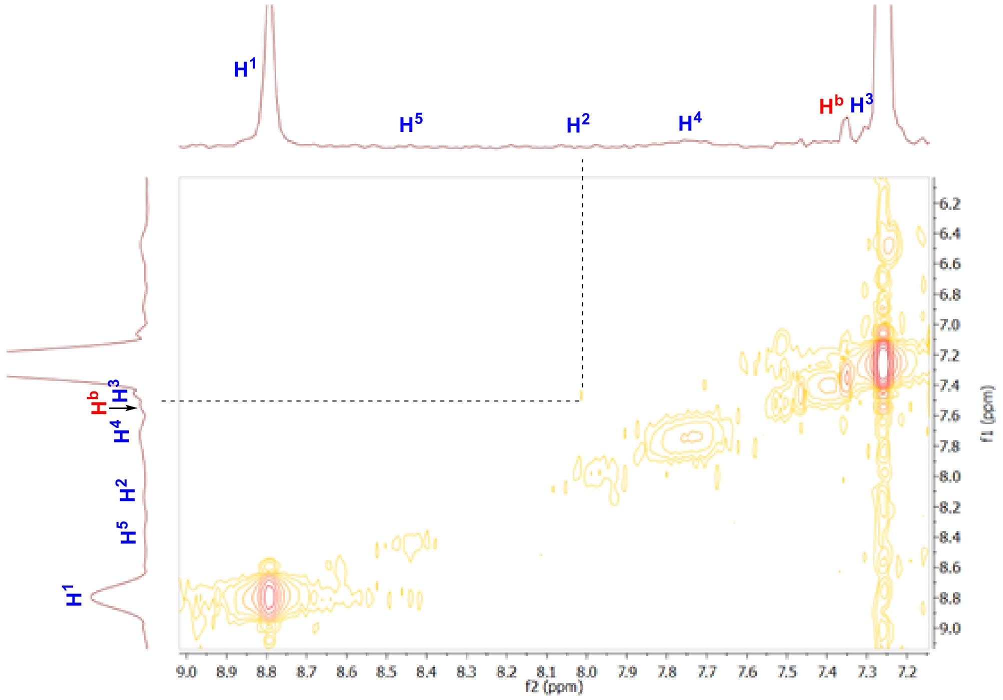

Supplementary Figure 15. NOESY spectrum (500 MHz, $CDCl_3$, 22 ℃) of a mixture of **6** and **9**.

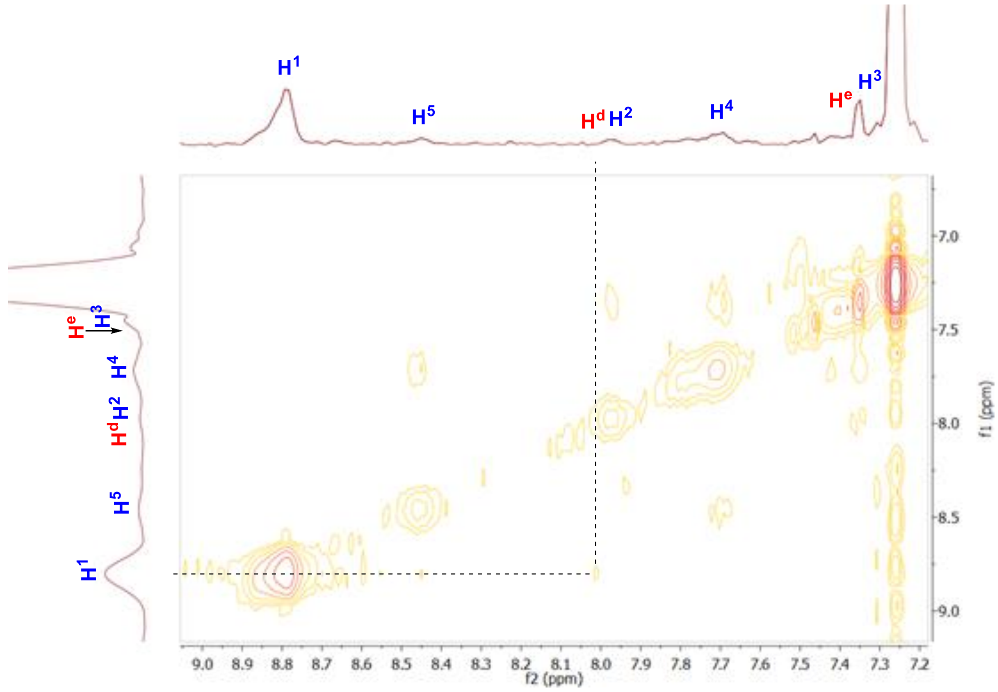

Supplementary Figure 16. NOESY spectrum (500 MHz, $CDCl_3$, 22 ℃) of a mixture of **6** and **10**.

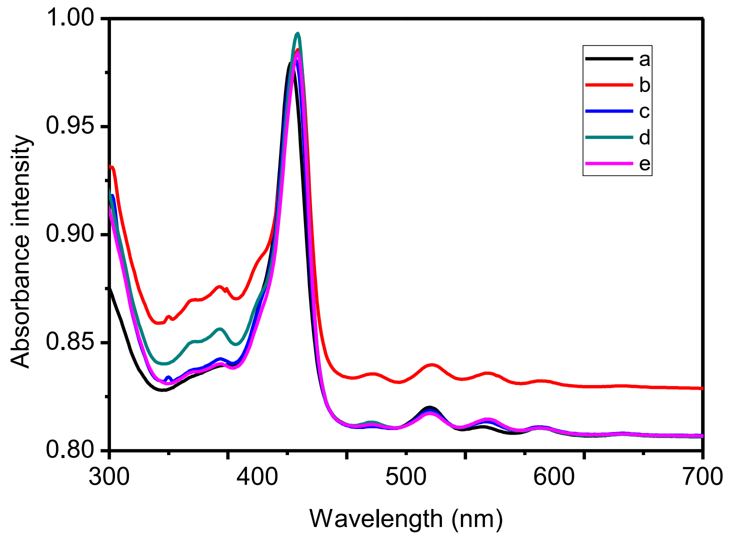

Supplementary Figure 17. The absorption spectral change of **5** upon the addition of guests in THF. **a** **5**. **b** **5** : **7** = 2 : 1 (mole ratio). **c** **5** : **7** : n-Bu_4NCl = 2 : 1 : 4 (mole ratio). **d** **5** : **8** = 2 : 1 (mole ratio). **e** **5** : **8** : n-Bu_4NCl = 2 : 1 : 4 (mole ratio). The concentration of **5** in all samples is 0.0067 mM.

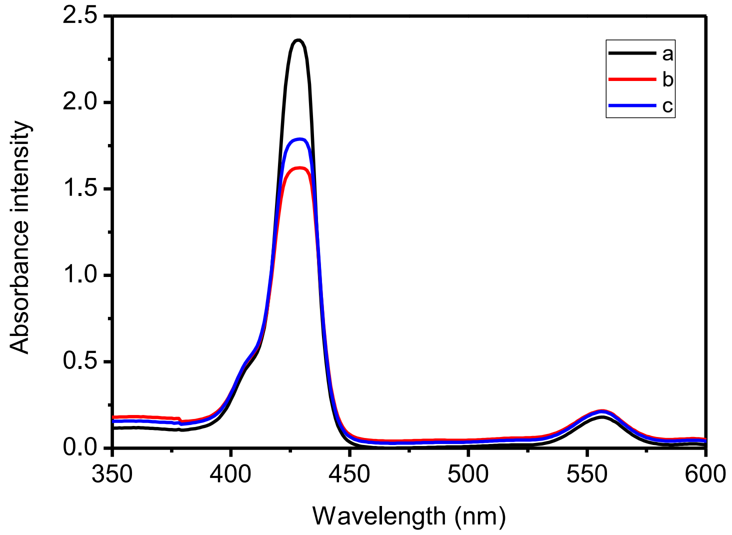

Supplementary Figure 18. The absorption spectral change of **6** upon the addition of guests in $CHCl_3$. **a** **6**. **b** **6** : **9** = 2 : 1 (mole ratio). **c** **6** : **10** = 3 : 1 (mole ratio). The concentration of **6** in all samples is 0.01 mM.

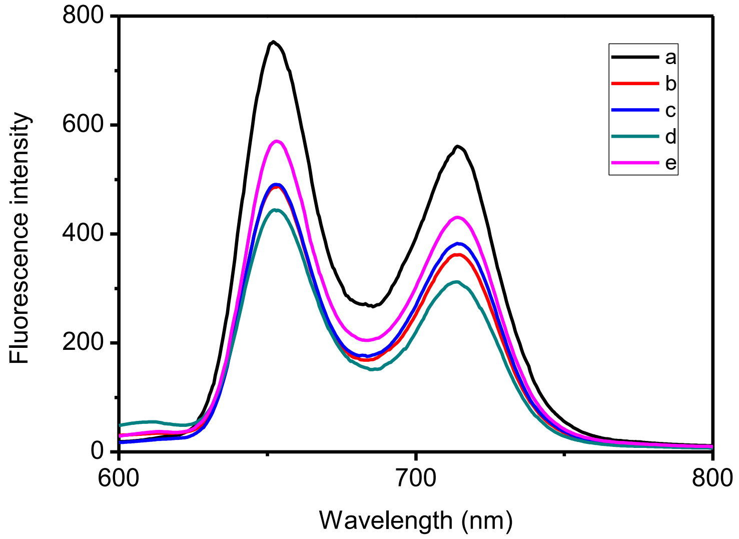

Supplementary Figure 19. Fluorescence intensity change of **5** upon the addition of guests, excited at 425 nm in THF. **a** **5**. **b** **5** : **7** = 2 : 1 (mole ratio). **c** **5** : **7** : *n*-Bu_4NCl = 2 : 1 : 4 (mole ratio). **d** **5** : **8** = 2 : 1 (mole ratio). **e** **5** : **8** : *n*-Bu_4NCl = 2 : 1 : 4 (mole ratio). The concentration of **5** in all samples is 0.0067 mM.

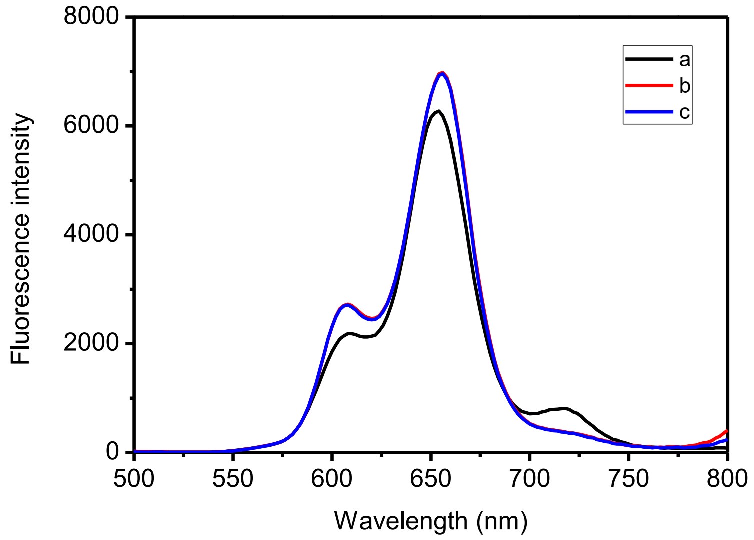

Supplementary Figure 20. Fluorescence intensity change of **6** upon the addition of guests, excited at 428 nm in $CHCl_3$. **a 6**. **b 6** : **9** = 2 : 1 (mole ratio). **c 6** : **10** = 3 : 1 (mole ratio). The concentration of **6** in all samples is 0.01 mM.

a

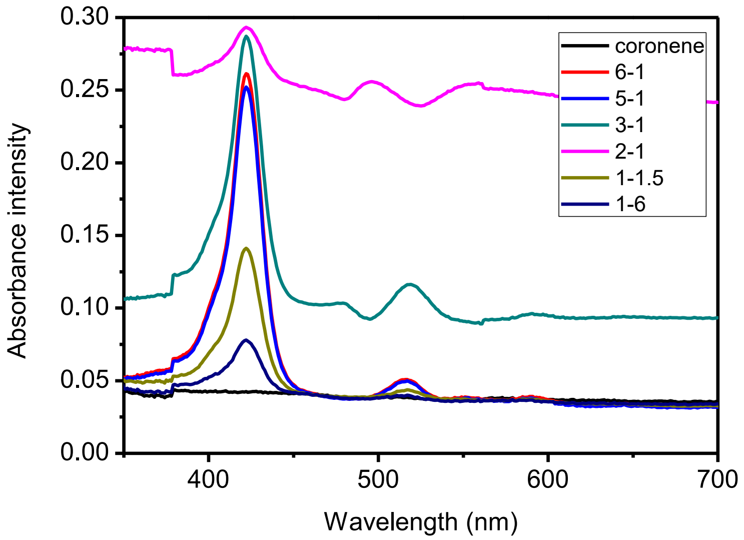

b

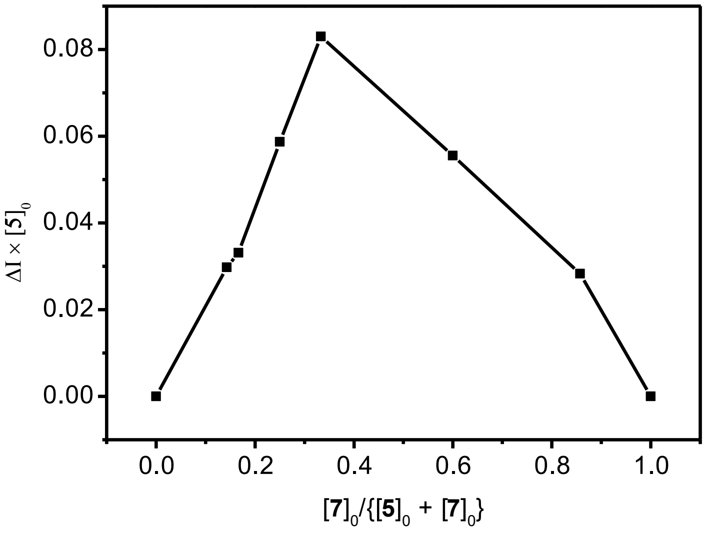

Supplementary Figure 21. UV-vis spectra and Job plot for $\mathbf{5}_2$•**7**. **a** UV-vis absorption of the mixture of **5** and **7** in toluene at different molar ratios. **b** Job plot showing a 2:1 stoichiometry of the complex between **5** and **7** by plotting $\Delta I \times [\mathbf{5}]_0$ (ΔI is the difference in absorption of **5** at 425 nm) against the mole fraction of **7** ($[\mathbf{7}]_0/\{[\mathbf{5}]_0 + [\mathbf{7}]_0\}$). $[\mathbf{5}]_0 + [\mathbf{7}]_0 = 1.0 \times 10^{-6}$ M. $[\mathbf{5}]_0$ and $[\mathbf{7}]_0$ are initial concentrations of **5** and **7**, respectively.

a

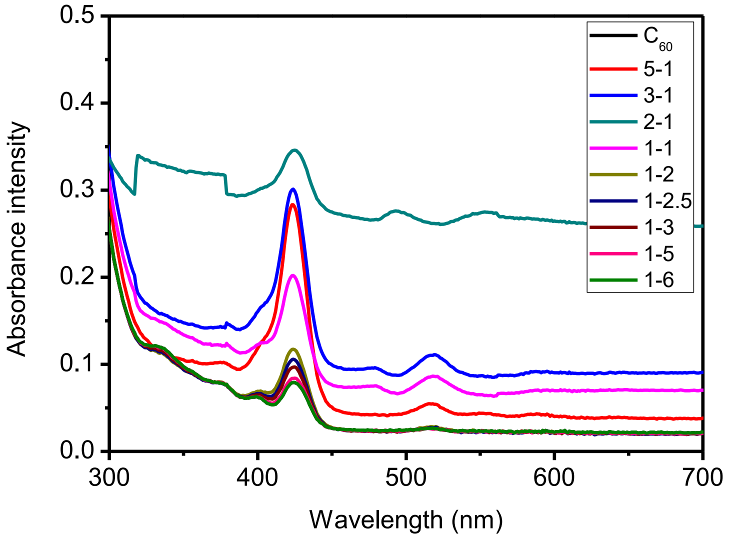

b

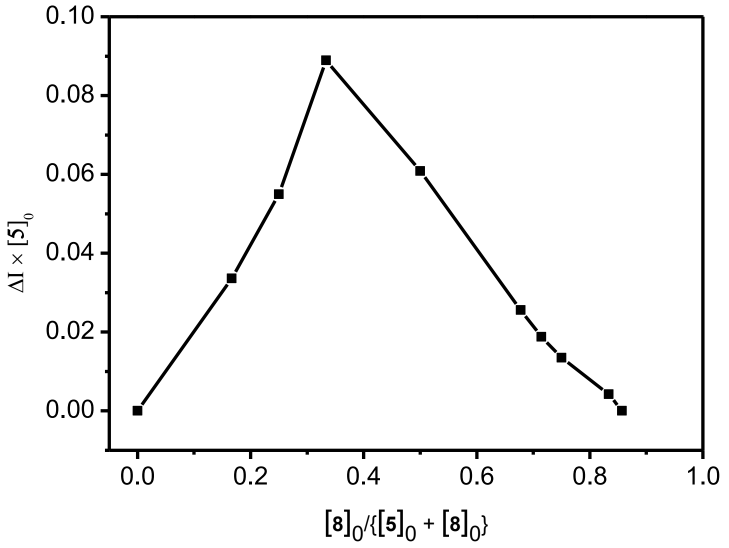

Supplementary Figure 22. UV-vis spectra and Job plot for $\mathbf{5}_2$•**8**. **a** UV-vis absorption of the mixture of **5** and **8** in toluene at different molar ratios. **b** Job plot showing a 2:1 stoichiometry of the complex between **5** and **8** by plotting $\Delta I \times [\mathbf{5}]_0$ (ΔI is the difference in absorption of **5** at 425 nm) against the mole fraction of **8** ($[\mathbf{8}]_0/\{[\mathbf{5}]_0 + [\mathbf{8}]_0\}$). $[\mathbf{5}]_0 + [\mathbf{8}]_0 = 1.0 \times 10^{-6}$ M. $[\mathbf{5}]_0$ and $[\mathbf{8}]_0$ are initial concentrations of **5** and **8**, respectively.

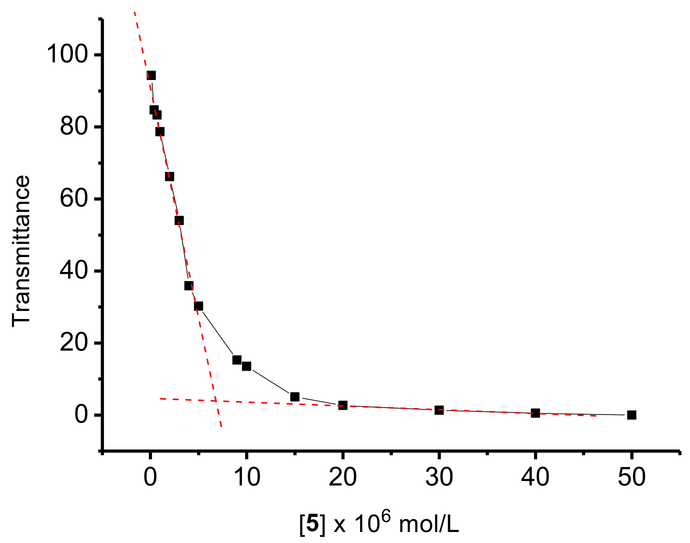

Supplementary Figure 23. CAC of the amphiphilic porphyrin **5** in aqueous solution measured via the UV-vis transmittance at different concentrations. The CAC of **5** in aqueous solution was estimated to be about 7.0 $\times 10^{-6}$ M.

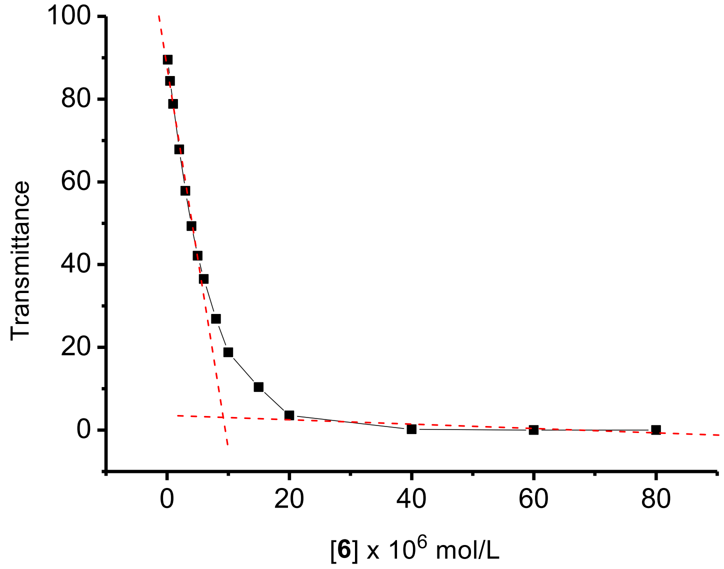

Supplementary Figure 24. CAC of the amphiphilic Zn-porphyrin **6** in aqueous solution measured via the UV-vis transmittance at different concentrations. The CAC of **6** in aqueous solution was estimated to be about 9.0 $\times 10^{-6}$ M.

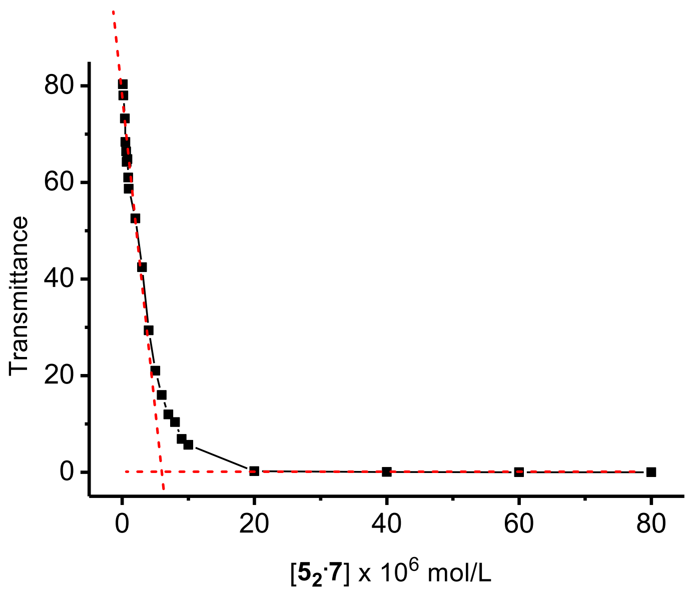

Supplementary Figure 25. CAC of $\mathbf{5}_2$•**7** in aqueous solution measured via the UV-vis transmittance at different concentrations. The CAC of $\mathbf{5}_2$•**7** in aqueous solution was estimated to be about 5.5 $\times 10^{-6}$ M.

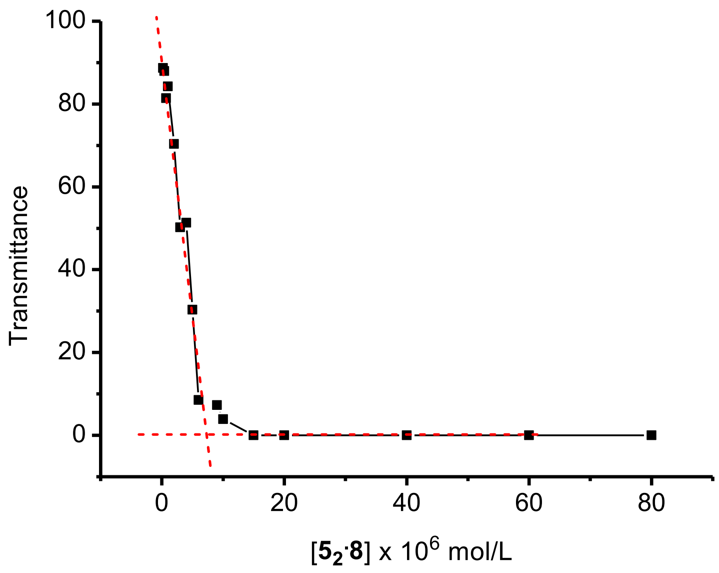

Supplementary Figure 26. CAC of $\mathbf{5}_2$•**8** in aqueous solution measured via the UV-vis transmittance at different concentrations. The CAC of $\mathbf{5}_2$•**8** in aqueous solution was estimated to be about 8.0 $\times 10^{-6}$ M.

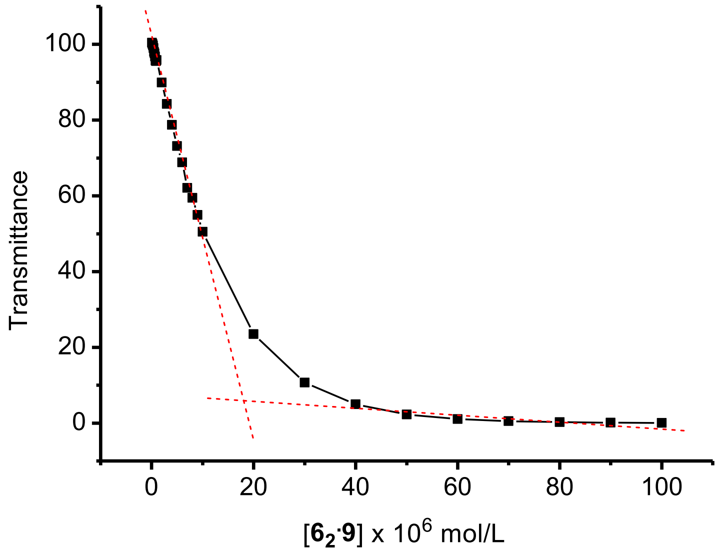

Supplementary Figure 27. CAC of $\mathbf{6_2}$•**9** in aqueous solution measured via the UV-vis transmittance at different concentrations. The CAC of $\mathbf{6_2}$•**9** in aqueous solution was estimated to be about 1.8×10^{-5} M.

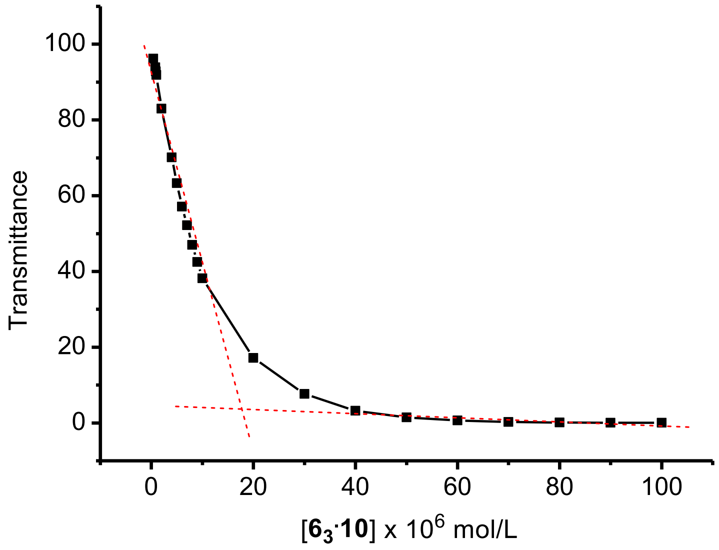

Supplementary Figure 28. CAC of $\mathbf{6_3}$•**10** in aqueous solution measured via the UV-vis transmittance at different concentrations. The CAC of $\mathbf{6_3}$•**10** in aqueous solution was estimated to be about 1.7×10^{-5} M.

a

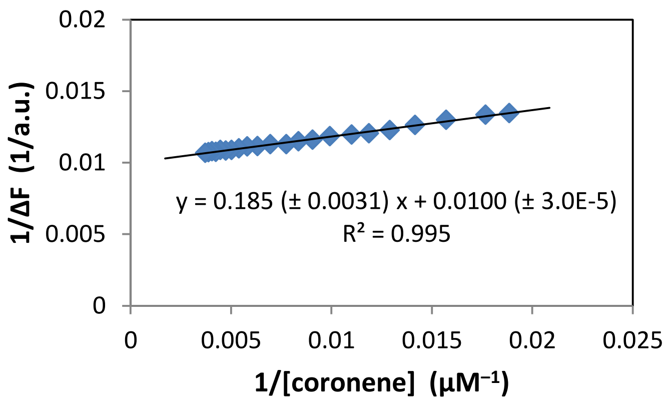

b

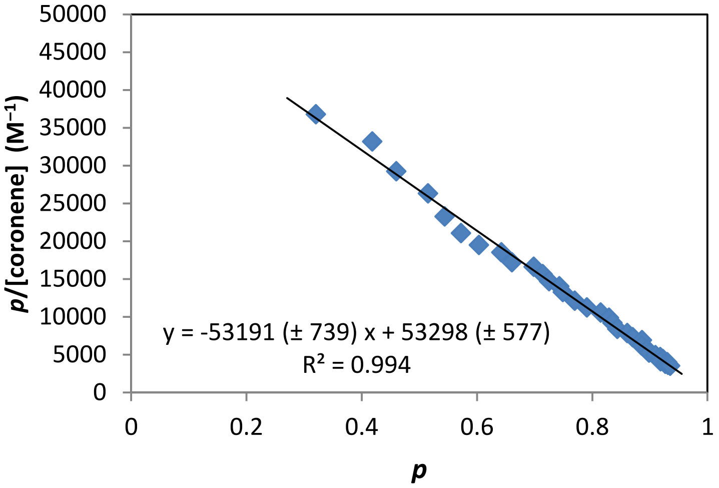

Supplementary Figure 29. Benesi-Hildebrand plot and Scatchard plot for **5**$_2$•**7**. **a** Benesi-Hildebrand plot for complexation of **5** with coronene (**7**) in THF at 22 ℃. ΔF_0, the difference in fluorescence intensity at 648 nm of **5** excited at λ = 422 nm, was determined as the y-intercept of a plot of $\Delta F = F_0 - F$ versus 1/[coronene]$_0$ in the high initial concentration range of coronene, wherein F_0 is the fluorescence intensity without the addition of coronene and F is the fluorescence intensity with the addition of coronene at different initial concentration, [coronene]$_0$; ΔF_0 = 1/0.0100 = 100 a.u.. **b** Scatchard plot for complexation of **5** with **7** in THF at 22 ℃. p = fraction of porphyrin units bound. $p = \Delta F/\Delta F_0$; ΔF is the observed fluorescence change with the addition of coronene at different initial concentration. The linear nature of this plot indicates that the complexation between **5** and **7** is statistical, that is, the two binding sites behaved independently. From the intercept and the slope of the Scatchard plot, the average association constant ($K_{av} = (K_1 + K_2)/2$) was determined to be 5.3 (±0.1) $\times 10^4$ M^{-1} for **5**$_2$•**7**. Since K_1/K_2 = 4:1 for statistical systems (K_1 = [**5**•**7**]/{[**5**][**7**]} and K_2 = [**5**$_2$•**7**]/{[**5**•**7**][**5**]}), K_1 and K_2 were calculated to be 8.5 (±0.2) $\times 10^4$ M^{-1} and 2.1 (±0.1) $\times 10^4$ M^{-1}, respectively.

a

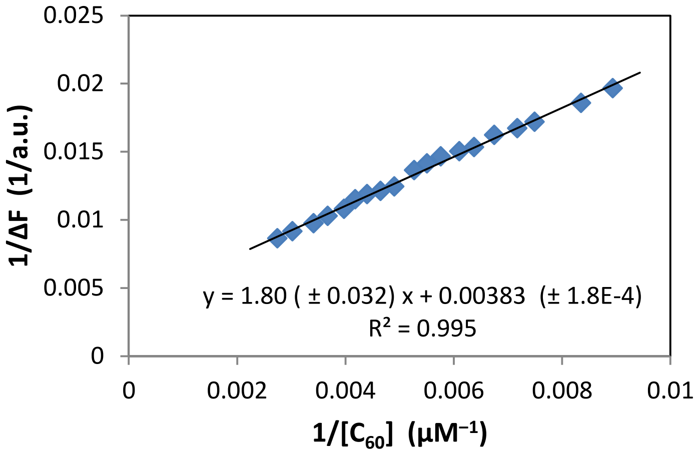

b

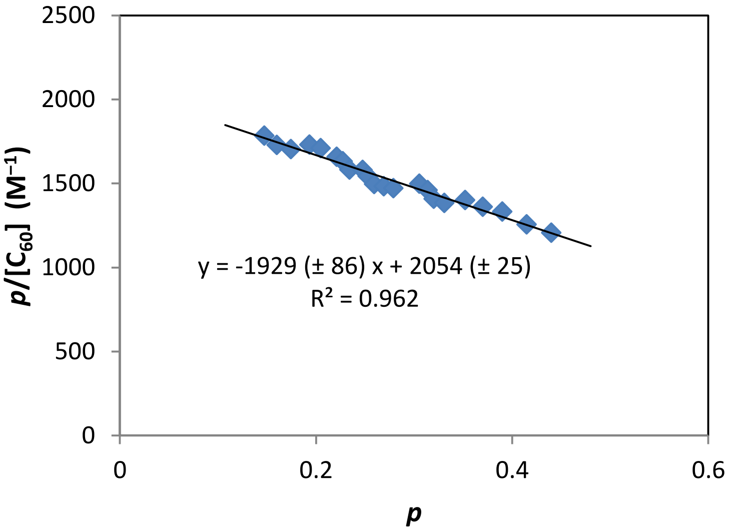

Supplementary Figure 30. Benesi-Hildebrand plot and Scatchard plot for **5**$_2$•**8**. **a** Benesi-Hildebrand plot for complexation of **5** with C_{60} (**8**) in toluene at 22 ℃. ΔF_0, the difference in fluorescence intensity at 648 nm of **5** excited at λ = 422 nm, was determined as the y-intercept of a plot of $\Delta F = F_0 - F$ versus $1/[C_{60}]_0$ in the high initial concentration range of C_{60}, wherein F_0 is the fluorescence intensity without the addition of C_{60} and F is the fluorescence intensity with the addition of C_{60} at different initial concentration, $[C_{60}]_0$; ΔF_0 = 1/0.00383 = 261 a.u.. **b** Scatchard plot for complexation of **5** with C_{60} in toluene at 22 ℃. p = fraction of porphyrin units bound. $p = \Delta F/\Delta F_0$; ΔF is the observed fluorescence change with the addition of C_{60} at different initial concentration. The linear nature of this plot indicates that the complexation between **5** and **8** is statistical, that is, the two binding sites behaved independently. From the intercept and the slope of the Scatchard plot, the average association constant ($K_{av} = (K_1 + K_2)/2$) was determined to be 2.0 (± 0.1) × 10^3 M^{-1} for **5**$_2$•**8**. Since K_1/K_2 = 4:1 for statistical systems (K_1 = [**5**•**8**]/{[**5**][**8**]} and K_2 = [**5**$_2$•**8**]/{[**5**•**8**][**5**]}), K_1 and K_2 were calculated to be 3.2 (± 0.2) × 10^3 M^{-1} and 8.0 (± 0.4) × 10^2 M^{-1}, respectively.

a

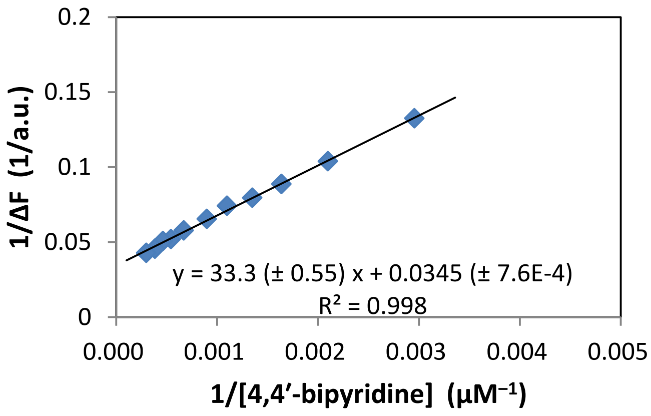

b

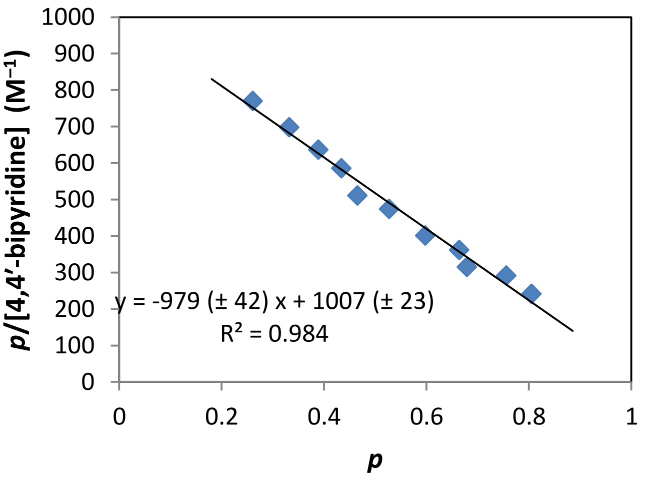

Supplementary Figure 31. Benesi-Hildebrand plot and Scatchard plot for **6**$_2$•**9**. **a** Benesi-Hildebrand plot for complexation of **6** with 4,4′-bipyridine (**9**) in THF at 22 ℃. ΔF_0, the difference in fluorescence intensity at 650 nm of **6** excited at λ = 422 nm, was determined as the y-intercept of a plot of $\Delta F = F_0 - F$ versus 1/[4,4′-bipyridine]$_0$ in the high initial concentration range of 4,4′-bipyridine, wherein F_0 is the fluorescence intensity without the addition of 4,4′-bipyridine and F is the fluorescence intensity with the addition of 4,4′-bipyridine at different initial concentration, [4,4′-bipyridine]$_0$; ΔF_0 = 1/0.0345 = 29.0 a.u.. **b** Scatchard plot for complexation of **6** with 4,4′-bipyridine in THF at 22 ℃. *p* = fraction of Zn-porphyrin units bound. $p = \Delta F/\Delta F_0$; ΔF is the observed fluorescence change with the addition of 4,4′-bipyridine at different initial concentration. The linear nature of this plot indicates that the complexation between **6** and **9** is statistical, that is, the two binding sites behaved independently. From the intercept and the slope of the Scatchard plot, the average association constant ($K_{av} = (K_1 + K_2)/2$) was determined to be 9.9 (± 0.4) × 10^2 M^{-1} for **6**$_2$•**9**. Since K_1/K_2 = 4:1 for statistical systems (K_1 = [**6**•**9**]/{[**6**][**9**]} and K_2 = [**6**$_2$•**9**]/{[**6**•**9**][**6**]}), K_1 and K_2 were calculated to be 1.6 (± 0.1) × 10^3 M^{-1} and 4.0 (± 0.2) × 10^2 M^{-1}, respectively.

a

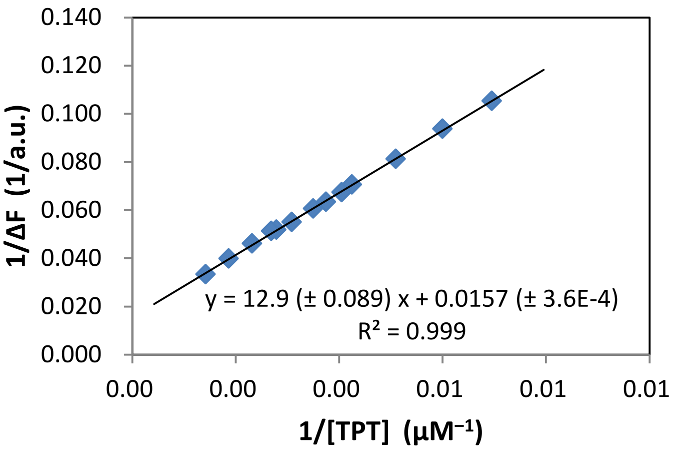

b

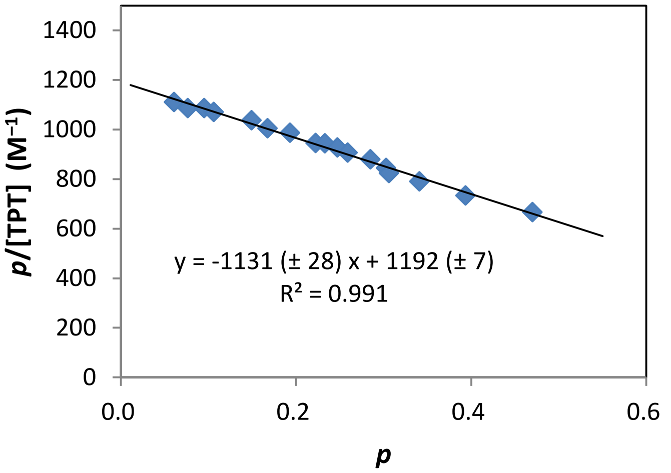

Supplementary Figure 32. Benesi-Hildebrand plot and Scatchard plot for **6**$_3$•**10**. **a** Benesi-Hildebrand plot for complexation of **6** with TPT (**10**) in THF at 22 ℃. ΔF_0, the difference in fluorescence intensity at 650 nm of **6** excited at λ = 422 nm, was determined as the y-intercept of a plot of $\Delta F = F_0 - F$ versus $1/[TPT]_0$ in the high initial concentration range of TPT, wherein F_0 is the fluorescence intensity without the addition of TPT and F is the fluorescence intensity with the addition of TPT at different initial concentration, $[TPT]_0$; $\Delta F_0 = 1/0.0157 = 63.7$ a.u.. **b** Scatchard plot for complexation of **6** with **10** in THF at 22 ℃. p = fraction of Zn-porphyrin units bound. $p = \Delta F/\Delta F_0$; ΔF is the observed fluorescence change with the addition of TPT at different initial concentration. The linear nature of this plot indicates that the complexation between **6** and **10** is statistical, that is, the three binding sites behaved independently. From the intercept and the slope of the Scatchard plot, the average association constant ($K_{av} = (K_1 + K_2 + K_3)/3$) was determined to be 1.2 ($\pm$0.1) $\times 10^3$ M^{-1} for **6**$_3$•**10**. Since $K_1/K_2/K_3$ = 9:3:1 for statistical systems (K_1 = [**6**•**10**]/{[**6**][**10**]}, K_2 = [**6**$_2$•**10**]/{[**6**•**10**][**6**]} and K_3 = [**6**$_3$•**10**]/{[**6**$_2$•**10**][**6**]}), K_1, K_2 and K_3 were calculated to be 2.4 ($\pm$0.1) $\times 10^3$ M^{-1}, 8.1 ($\pm$0.1) $\times 10^2$ M^{-1} and 2.7 ($\pm$0.1) $\times 10^2$ M^{-1}, respectively.

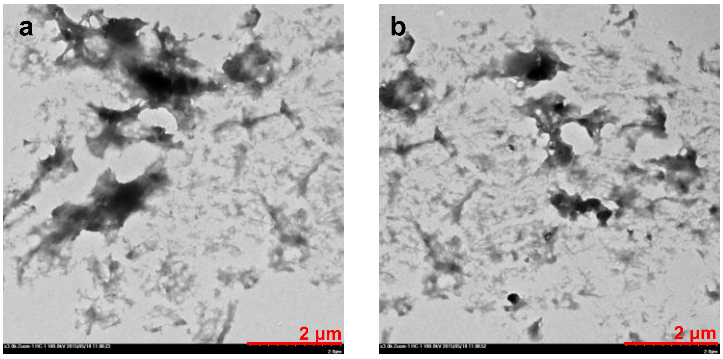

Supplementary Figure 33. TEM images of **5** in aqueous solution. The concentration of the samples is 1 mM.

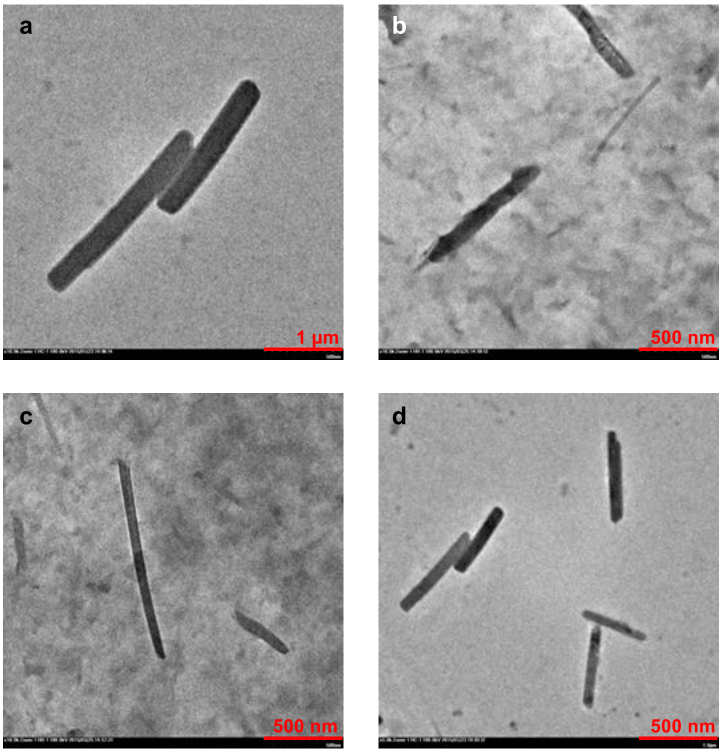

Supplementary Figure 34. TEM images of **5_2•7** in aqueous solution. The concentration of the samples is 1 mM.

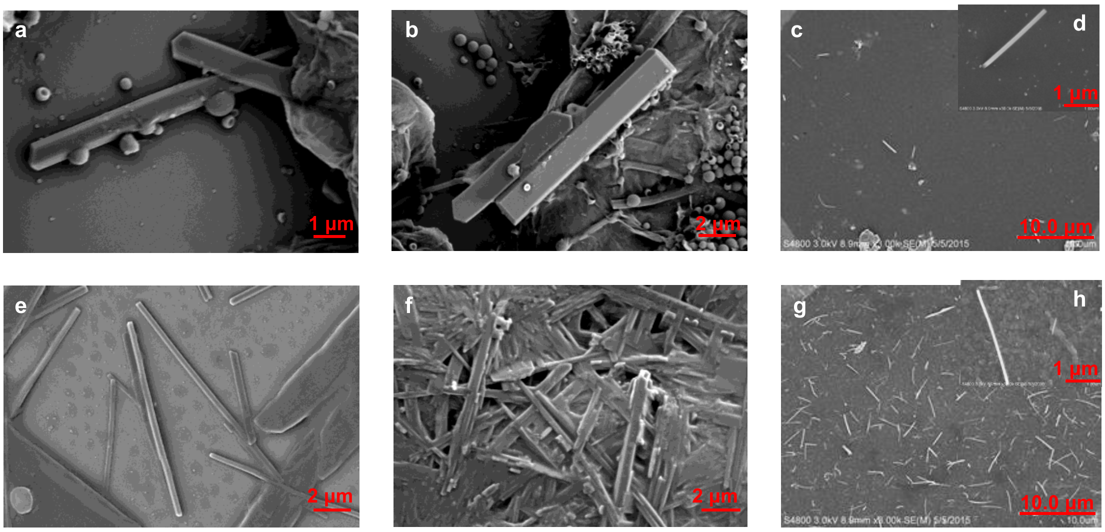

Supplementary Figure 35. SEM images of $\mathbf{5_2{\bullet}7}$. **a** and **b** SEM images of $\mathbf{5_2{\bullet}7}$ at 1 mM in aqueous solution without addition of Cl^-. **c** and **d** SEM images of $\mathbf{5_2{\bullet}7}$ at 0.1 mM in aqueous solution without addition of Cl^-. **e** and **f** SEM images of $\mathbf{5_2{\bullet}7}$ at 1 mM in aqueous solution with the addition of *n*-Bu_4NCl. **g** and **h** SEM images of $\mathbf{5_2{\bullet}7}$ at 0.1 mM with the addition of *n*-Bu_4NCl in aqueous solution.

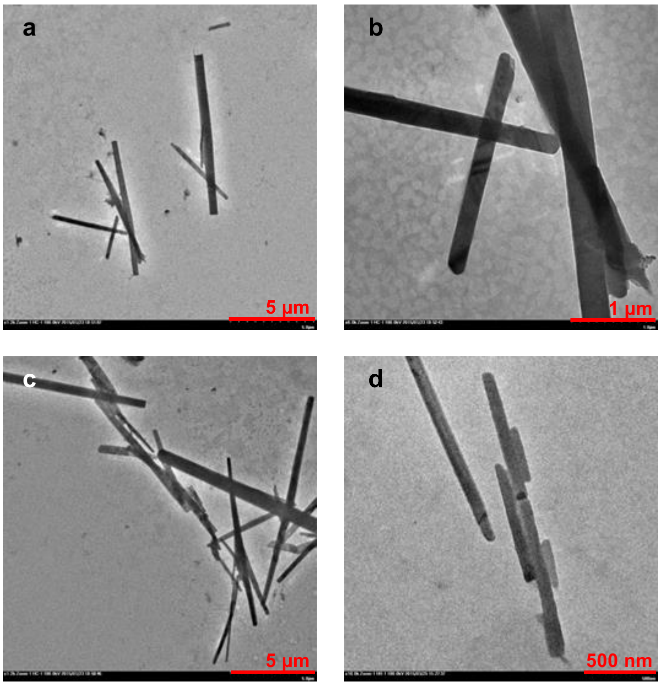

Supplementary Figure 36. TEM images of $\mathbf{5_2{\bullet}7}$ with the addition of *n*-Bu_4NCl in aqueous solution. The concentration of the samples is 1 mM.

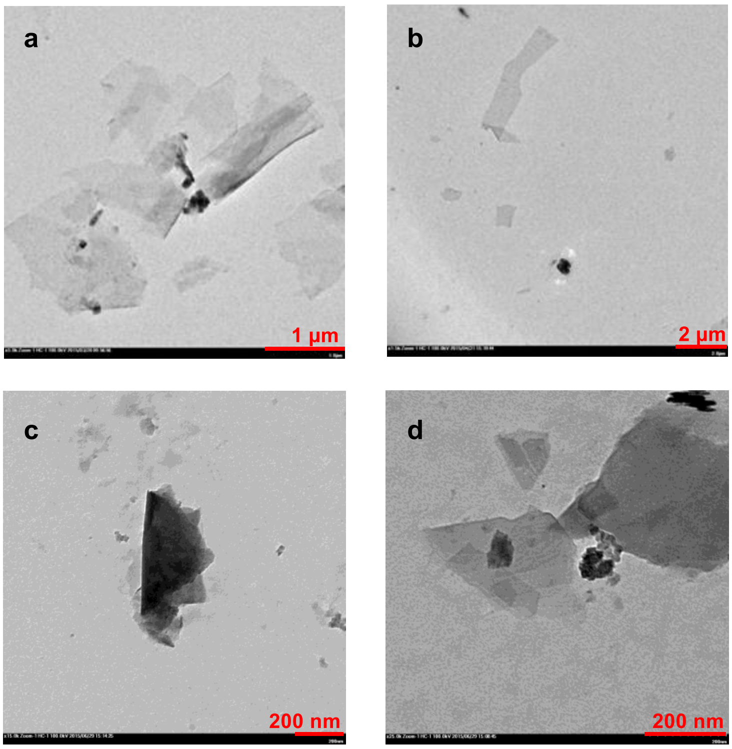

Supplementary Figure 37. TEM images of $\mathbf{5_2{\bullet}8}$ in aqueous solution. The concentration of the samples is 0.1 mM.

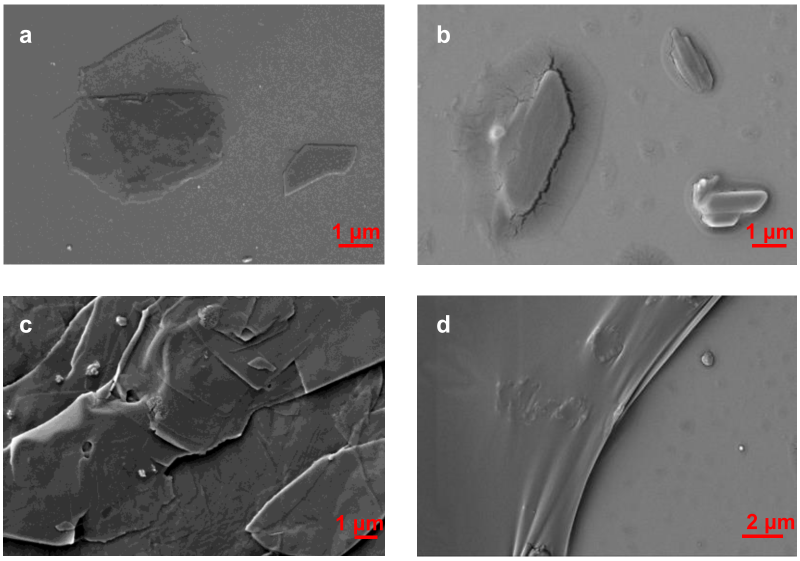

Supplementary Figure 38. SEM images of $\mathbf{5_2{\bullet}8}$. **a** and **b** SEM images of $\mathbf{5_2{\bullet}8}$ at 0.1 mM without *n*-Bu_4NCl in aqueous solution. **c** and **d** SEM images of $\mathbf{5_2{\bullet}8}$ at 1 mM with the addition of *n*-Bu_4NCl in aqueous solution.

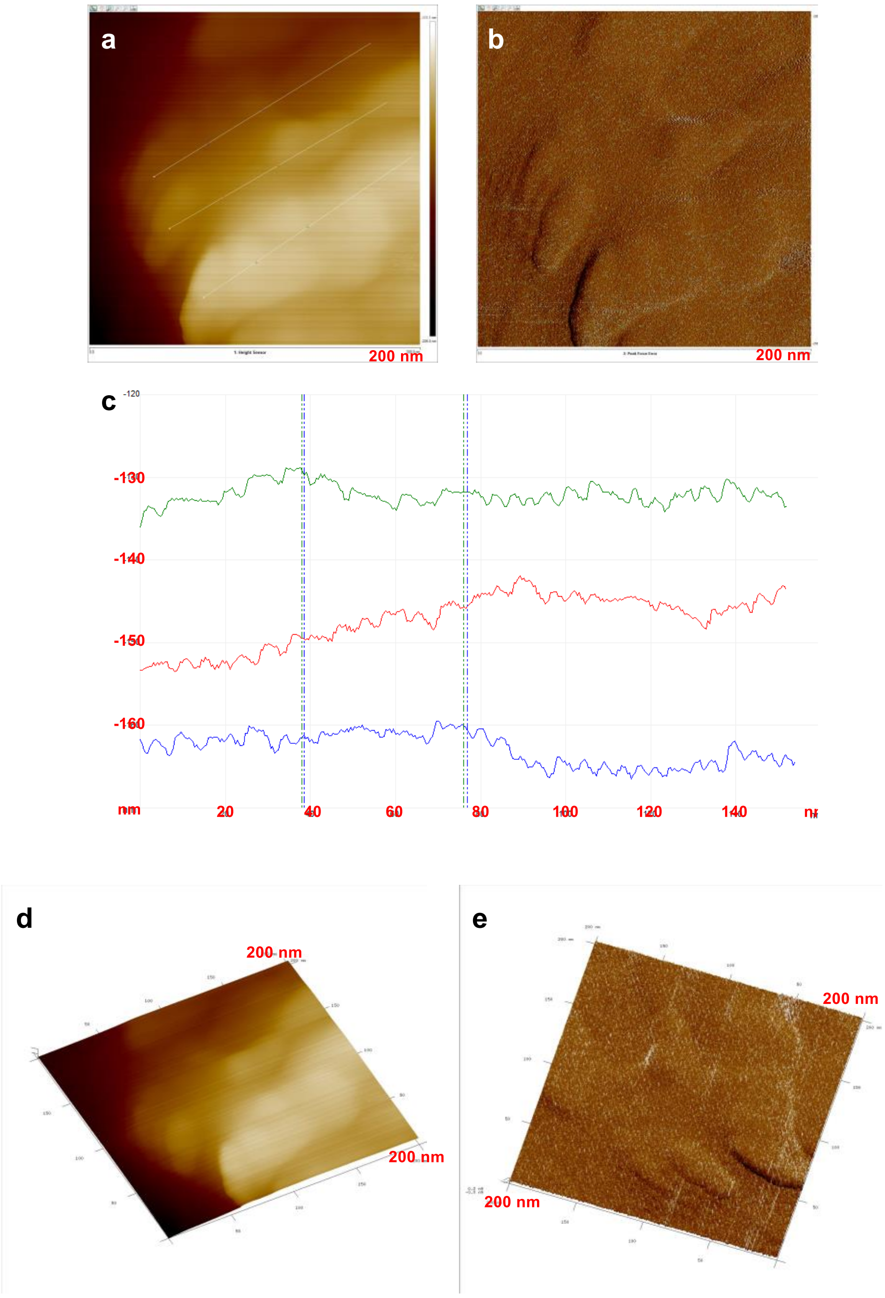

Supplementary Figure 39. AFM images of **5** upon the addition of **8** in aqueous solution. The concentration of the samples is 0.1 mM.

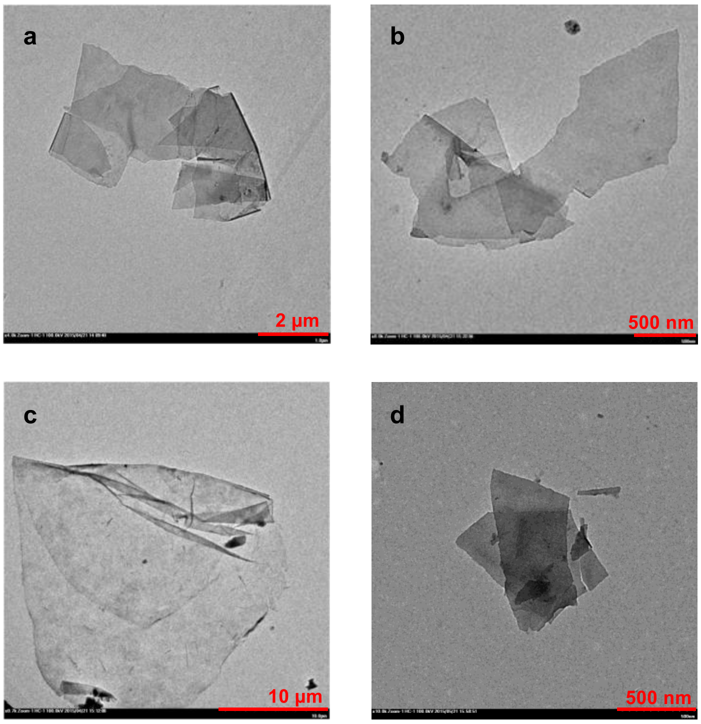

Supplementary Figure 40. TEM images of $\mathbf{5_2{\bullet}8}$ with the addition of n-Bu_4NCl in aqueous solution. The concentration of the samples is 0.1 mM.

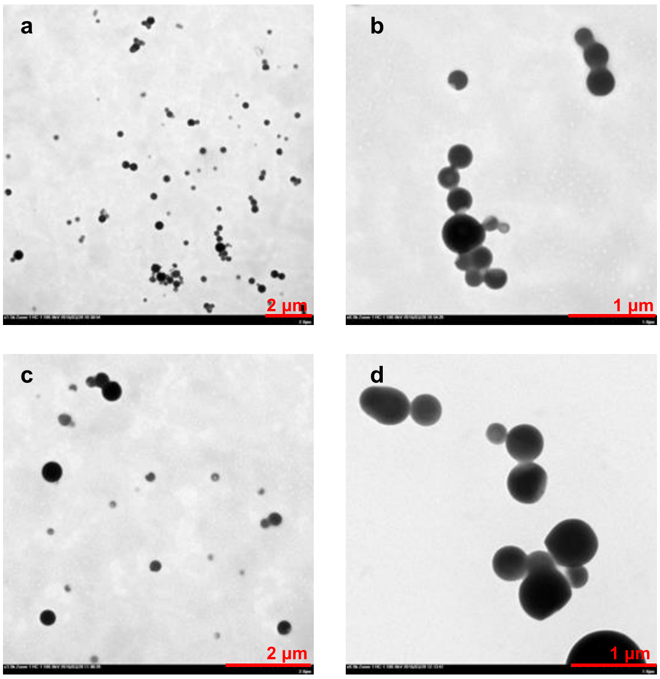

Supplementary Figure 41. TEM images of $\mathbf{6_2{\bullet}9}$ in aqueous solution. The concentration of the samples is 1 mM.

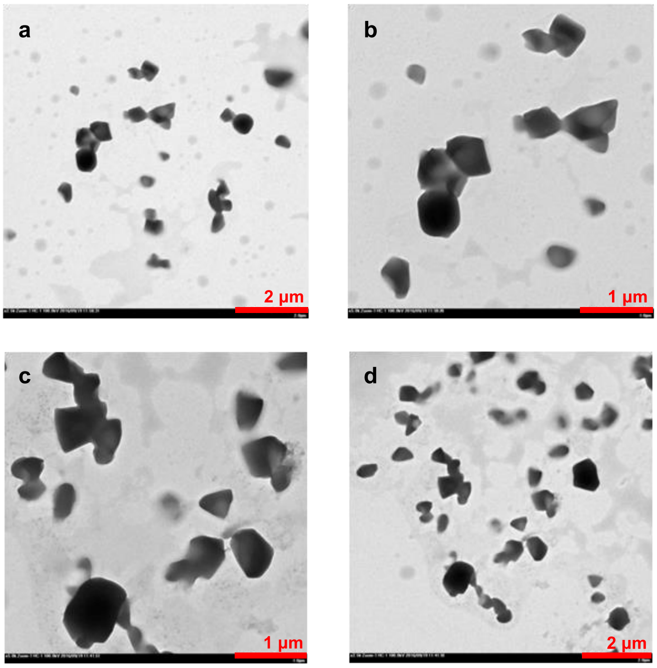

Supplementary Figure 42. TEM images of $\mathbf{6_2{\bullet}9}$ upon the addition of *n*-Bu_4NCl in aqueous solution. The concentration of the samples is 1 mM.

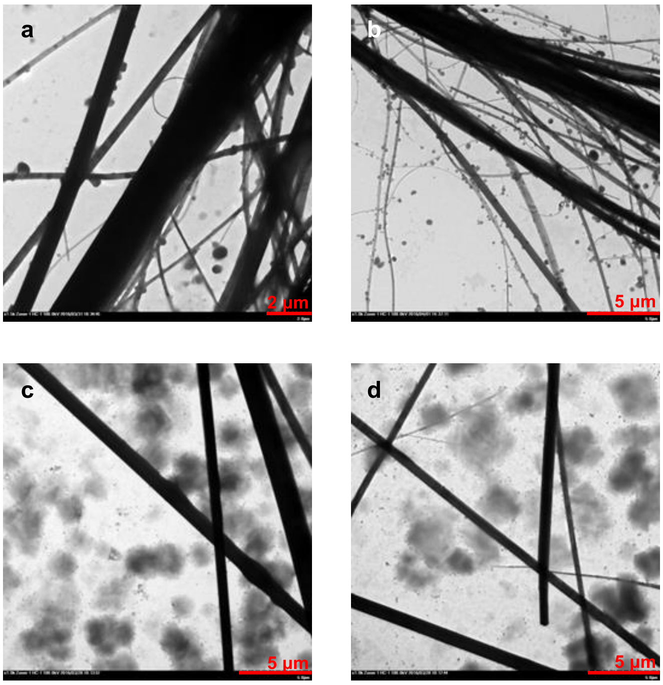

Supplementary Figure 43. TEM images of $\mathbf{6_3{\bullet}10}$ in aqueous solution. The concentration of the samples is 1 mM.

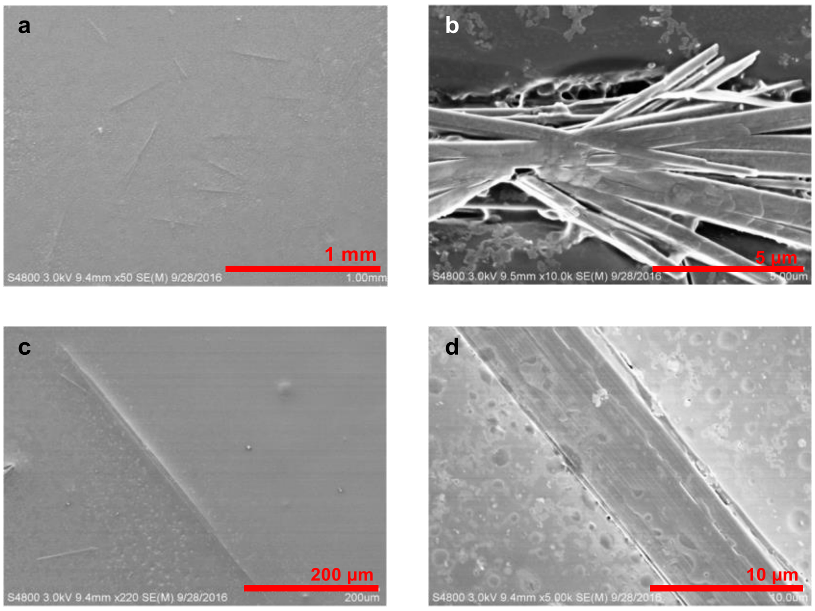

Supplementary Figure 44. SEM images of **6_3•10** in aqueous solution. The concentration of the samples is 1 mM.

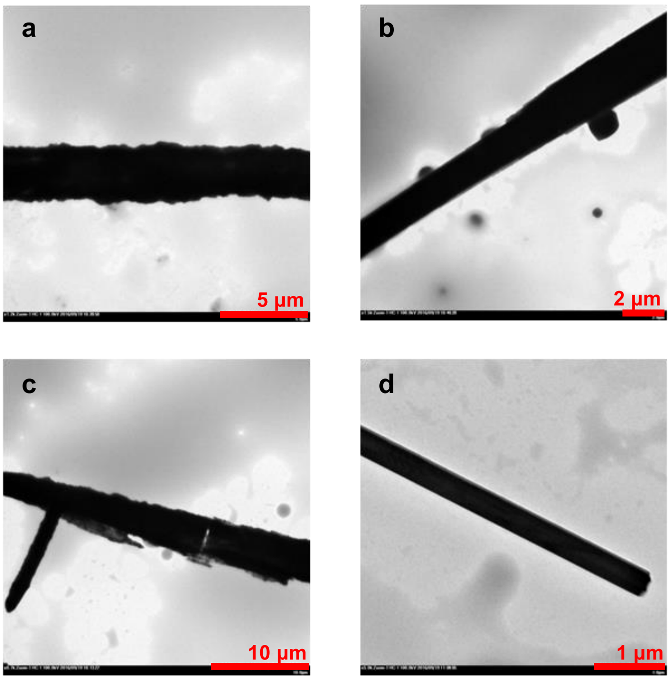

Supplementary Figure 45. TEM images of **6_3•10** upon the addition of *n*-Bu_4NCl in aqueous solution. The concentration of the samples is 1 mM.

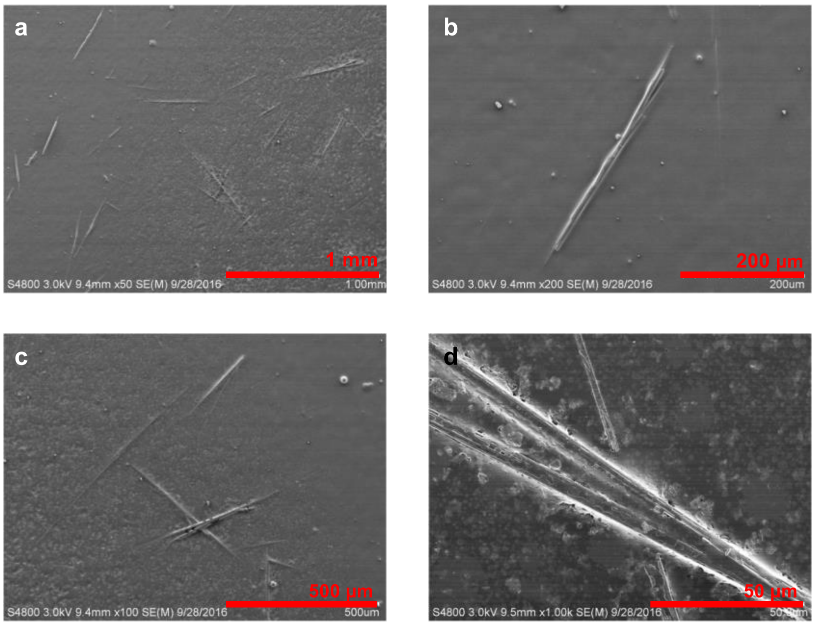

Supplementary Figure 46. SEM images of **6_3•10** upon the addition of *n*-Bu_4NCl in aqueous solution. The concentration of the samples is 1 mM.

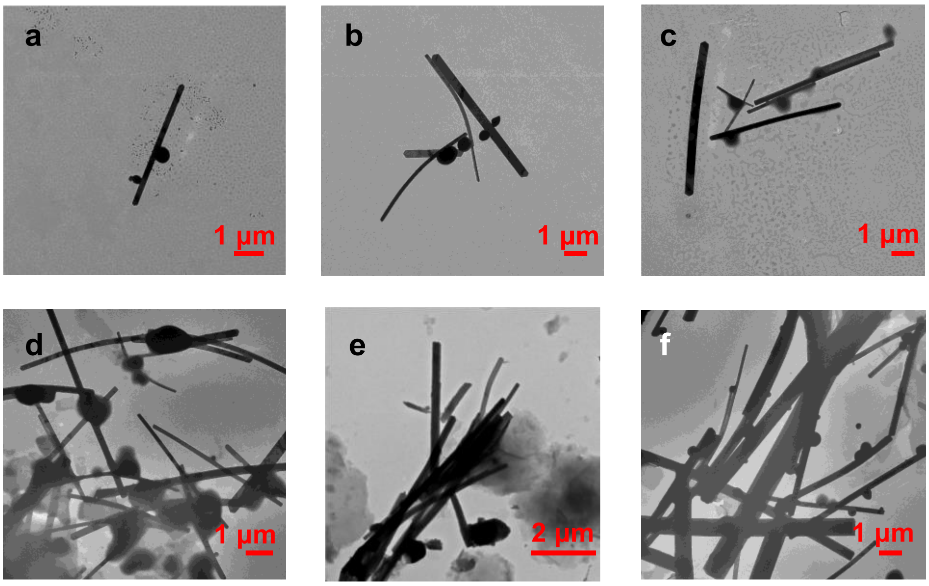

Supplementary Figure 47. TEM images of **5_2•7**. **a** After 3 days. **b** After 7 days. **c** Sample prepared at 60 ℃. **d** With the addition of *n*-Bu_4NCl after 3 days. **e** With the addition of *n*-Bu_4NCl after 7 days. **f** Sample prepared at 60 ℃ with the addition of *n*-Bu_4NCl. The concentration of the samples is 1 mM.

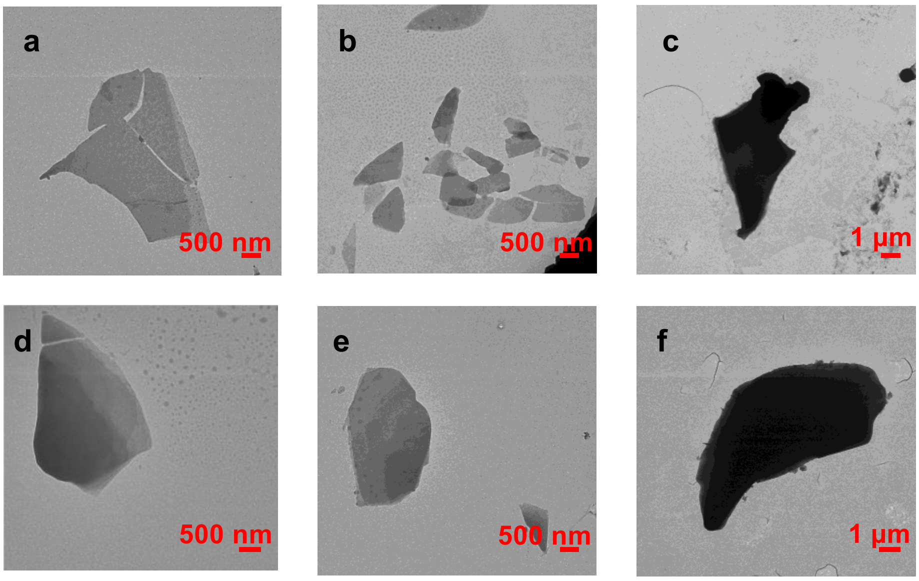

Supplementary Figure 48. TEM images of **5_2•8**. **a** After 3 days. **b** After 7 days. **c** Sample prepared at 60 ℃. **d** With the addition of *n*-Bu_4NCl after 3 days. **e** With the addition of *n*-Bu_4NCl after 7 days. **f** Sample prepared at 60 ℃ with the addition of *n*-Bu_4NCl. The concentration of the samples is 1 mM.

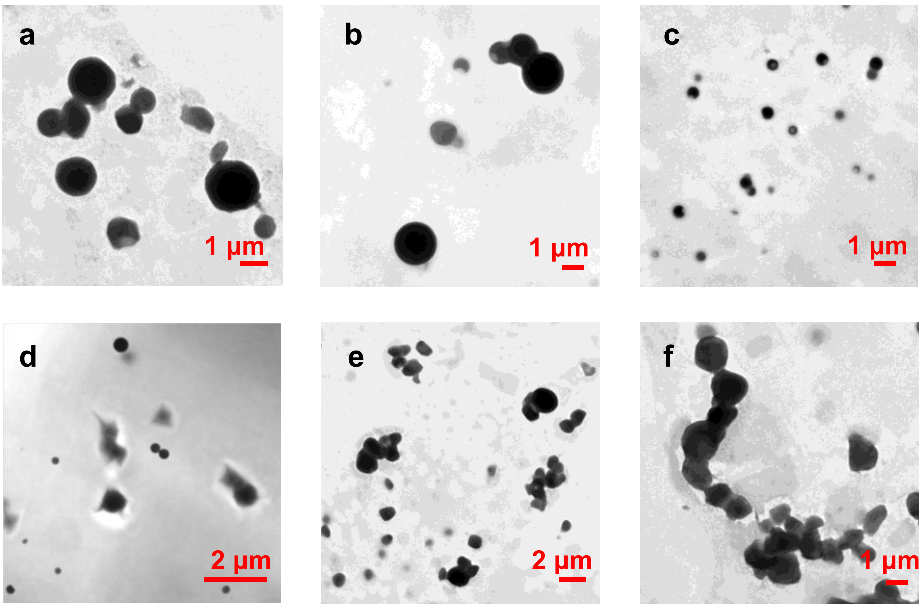

Supplementary Figure 49. TEM images of **6_2•9**. **a** After 3 days. **b** After 7 days. **c** Sample prepared at 60 ℃. **d** With the addition of *n*-Bu_4NCl after 3 days. **e** With the addition of *n*-Bu_4NCl after 7 days. **f** Sample prepared at 60 ℃ with the addition of *n*-Bu_4NCl. The concentration of the samples is 1 mM.

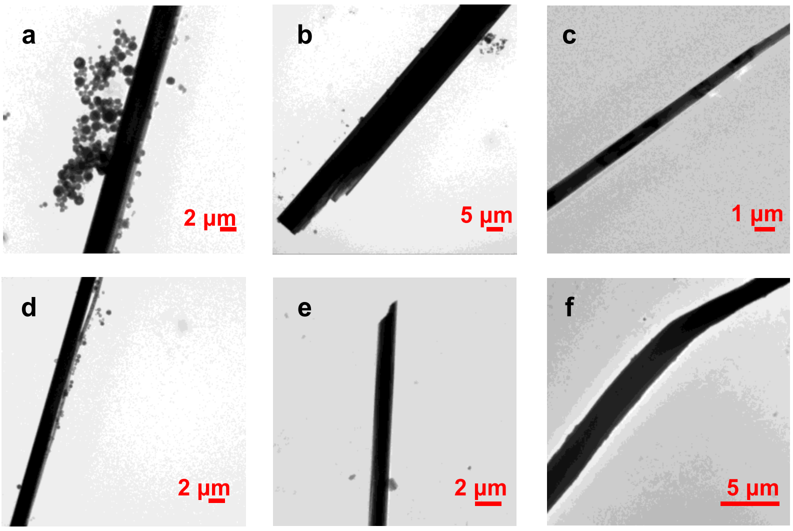

Supplementary Figure 50. TEM images of **6_3•10**. **a** After 3 days. **b** After 7 days. **c** Sample prepared at 60 ℃. **d** With the addition of *n*-Bu_4NCl after 3 days. **e** With the addition of *n*-Bu_4NCl after 7 days. **f** Sample prepared at 60 ℃ with the addition of *n*-Bu_4NCl. The concentration of the samples is 1 mM.

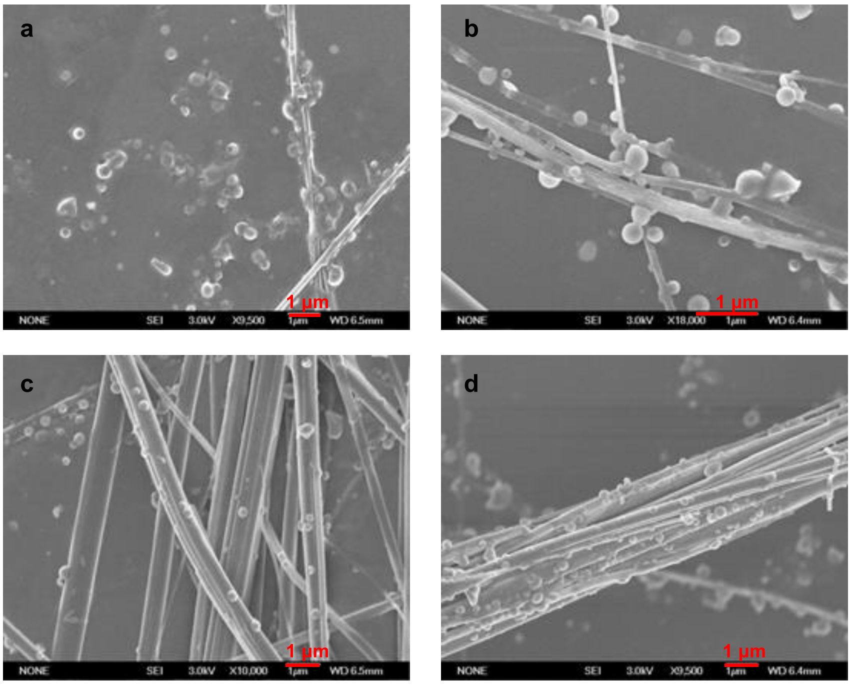

Supplementary Figure 51. SEM images of the self-assembly process: Structural changes during the self-assembly of **6_3•10** represented with SEM images investigated at different stages. **a** The nanoparticles aggregated into nanowires. **b** The nanoparticles adhered to the nanowires gradually. **c** Microwires with smooth surface formed. **d** The macrowires bonded together to form stable wire bundles eventually.
